# In Silico ADME Methods Used in the Evaluation of Natural Products

**DOI:** 10.3390/pharmaceutics17081002

**Published:** 2025-07-31

**Authors:** Robert Ancuceanu, Beatrice Elena Lascu, Doina Drăgănescu, Mihaela Dinu

**Affiliations:** 1 Faculty of Pharmacy, Department of Pharmaceutical Botany and Cell Biology, Carol Davila University of Medicine and Pharmacy, 050474 București, Romania; robert.ancuceanu@umfcd.ro (R.A.); mihaela.dinu@umfcd.ro (M.D.); 2 Faculty of Pharmacy, Department of Pharmaceutical Physics and Informatics, Carol Davila University of Medicine and Pharmacy, 050474 București, Romania

**Keywords:** ADME, in silico methods, natural compounds, quantum mechanics, molecular docking, pharmacophore modeling, QSAR, molecular dynamics, PBPK

## Abstract

The pharmaceutical industry faces significant challenges when promising drug candidates fail during development due to suboptimal ADME (absorption, distribution, metabolism, excretion) properties or toxicity concerns. Natural compounds are subject to the same pharmacokinetic considerations. In silico approaches offer a compelling advantage—they eliminate the need for physical samples and laboratory facilities, while providing rapid and cost-effective alternatives to expensive and time-consuming experimental testing. Computational methods can often effectively address common challenges associated with natural compounds, such as chemical instability and poor solubility. Through a review of the relevant scientific literature, we present a comprehensive analysis of in silico methods and tools used for ADME prediction, specifically examining their application to natural compounds. Whereas we focus on identifying the predominant computational approaches applicable to natural compounds, these tools were developed for conventional drug discovery and are of general use. We examine an array of computational approaches for evaluating natural compounds, including fundamental methods like quantum mechanics calculations, molecular docking, and pharmacophore modeling, as well as more complex techniques such as QSAR analysis, molecular dynamics simulations, and PBPK modeling.

## 1. Introduction

Many promising drug candidates fail to reach the market due to poor ADME characteristics (how the drug is absorbed, transported throughout the body, metabolized, and excreted) and safety concerns about toxicity. When these problems are uncovered late in drug development, pharmaceutical companies risk large financial losses since they have already invested heavily in clinical trials and other costly research processes. To address this dilemma, the pharmaceutical industry has significantly changed its strategy in recent decades. Companies are increasingly performing extensive ADMET (absorption, distribution, metabolism, excretion, and toxicity) screening considerably earlier in the drug discovery process. This early screening technique identifies and eliminates problematic compounds before they enter costly development phases, thereby saving money and boosting medication development efficiency [1,2]. The presence of pan-assay interference compounds (PAINS) is an additional significant concern. Deceptive findings in research tests can be generated by these challenging compounds, which appear to be active against a diverse array of targets when they are not. It is a significant waste of time and money to investigate these “frequent hitters” in the context of research and development [2,3].

Compared to synthetic molecules, natural compounds possess unique properties that influence drug discovery. They are more structurally diverse and complex; tend to be larger; contain a greater amount of oxygen (and less nitrogen, sulfur and halogens), more chiral centers, and less aromatic rings; and are more water-soluble. This provides them with a distinctive potential as drugs, even when they do not adhere to the conventional principles for drug-like properties, such as Lipinski’s rule of five [4,5]. Nevertheless, the utilization of natural compounds in the drug discovery process is hindered by a number of obstacles. It can be challenging to test natural extracts, identify the active constituents in complex mixtures, obtain sufficient material from nature, and protect discoveries with patents. The pharmaceutical industry has experienced a decline in drug discovery that concentrates on natural products as a result of these obstacles [4,6].

Moreover, the discovery and development of natural product-based drugs are being substantially improved by recent developments in biosynthetic engineering technologies and chemical synthesis, as well as “smart screening” techniques or the use of robotic equipment in chemical separations and developments in structural analysis. Complex natural compound scaffolds that were previously considered inaccessible can now be optimized with these technologies. This advancement enables the enrichment of screening libraries with natural products, hybrids derived from natural products, natural product analogs, and natural products inspired molecules, as well as superior structure functionalization approaches, including late-stage functionalization, for the optimization of natural product leads [4,6,7,8]. In addition to their potential applications in drug discovery, numerous bioactive natural compounds are essential components of our daily diets. Gaining an understanding of their properties is essential for the advancement of human health, as it facilitates the creation of enhanced foods, such as functional foods and dietary supplements [9].

Many of the challenges applicable in the understanding of the pharmacological or biological properties of natural compounds are also relevant when exploring their ADME properties. For instance, often the available quantities of natural products are limited, and while numerous plant-derived natural products have been isolated and characterized, the amounts available are frequently insufficient for comprehensive ADME testing [10]. Using in silico methods from this point of view has a great advantage as they require no physical sample (not even picograms are necessary once the structural formula is available) or laboratory infrastructure. In addition (and this is an aspect relevant for all products of pharmaceutical interest, irrespective of their origin), the experimental assessment of the ADME properties of a substance is costly and time consuming [11], whereas the use of in silico tools is usually very cheap.

Natural compounds are often highly sensitive to environmental factors such as high temperature, moisture, intense light, oxygen, or pH variations. They may also be volatile or react with other substances and stability issues result in limited shelf-life, making it difficult to develop stable commercial products that maintain their essential properties over time [12]. Furthermore, many natural compounds may be degraded by stomach acid or undergo extensive metabolism in the liver before reaching their target sites in the body (first-pass metabolism) [13]. This is also a serious issue in assessing their ADME features in animal experiments, whereas using very cheap but valid in silico tools is very useful. An additional challenge for exploring ADME properties in wet lab experiments consists of the low aqueous solubility of natural compounds [14], which limits the ability to effectively deliver the compound to the biological system examined. Moreover, the growing need to minimize animal use in medical development and research highlights the increasing significance of in silico tools [15,16].

In this paper, we have proposed a synthesis of the scientific literature in order to understand the in silico methods and tools available for the prediction of ADME properties, with a focus on those that have been used to evaluate natural compounds. In this context, in silico methods are understood as computational methods used to explore scientific questions (including those related to PK) in the absence of physical experimentation; by in silico tools we understand algorithms and programs built to simulate, analyze, and make “predictions” about biological, chemical, and physical systems. We were interested in understanding which of the available methods and tools have been most frequently used in this field, although such methods and tools have been developed for drug discovery purposes, in general, and not specifically for natural products. We focus here exclusively on ADME (Absorption, Distribution, Metabolism, and Excretion) rather than ADMET. While both abbreviations are commonly used, our emphasis on ADME is related to its direct relevance to pharmacokinetics. The ‘T’ in ADMET primarily addresses safety concerns (a rather distinct field of study) and this limited focus on ADME allows for a clearer focus on the pharmacokinetic aspects of drug development.

This review will begin by outlining the primary methods used for ADME predictions and their applications for natural compounds. Subsequently, we will dedicate two separate sections to discussing the limitations and challenges associated with each method, as well as their performance in comparison to experimental data. We will conclude with a brief overview of the main in silico ADME tools that have been utilized to date in the field of natural compounds.

## 2. In Silico Methods Available for ADME Predictions

### 2.1. Quantum Mechanics (QM) and Molecular Mechanics (MM) Methods

Early studies of how drugs interact with receptors attempted to use quantum mechanics, but these calculations were limited because they required intensive computational resources. Thanks to significant advances in computer speed and new software, quantum mechanics calculations are now used more regularly to study drug-related problems (e.g., exploring enzyme–inhibitor interactions [17], predicting reactivity and stability [18], predicting routes of biotransformation [19], etc.). In the past three decades, such calculations have become much more common in studying ADME properties [20].

Cytochrome P450 (CYP) is a large group of enzymes, responsible for the biotransformation of about three quarters of drugs that subject to metabolism before elimination. Only four of these enzymes (CYP3A4, CYP2D6, CYP2C9, and CYP2C19) are estimated to be responsible for about 80% of all drug metabolisms mediated by the CYP family [21]. To better understand the molecular mechanisms involved in their functioning, P450cam, a CYP enzyme of bacterial origins, is commonly employed. This enzyme, *inter alia*, catalyzes the metabolism of camphor, a well-known natural compound, through 5-exo-hydroxylation. Simulations using quantum mechanics/molecular mechanics (QM/MM) on P450cam have resulted in controversial statements about the enzyme’s reactivity and its mechanisms of reaction, indicating that a propionate sidechain on the heme group undergoes partial oxidation and plays a key role catalysis. There have been attempts to reconcile multiple inconsistencies between theoretical and experimental data about this reaction. J. Zurek et al. (2006), using QM/MM, demonstrated that the heme propionates are not involved in the catalytic process [22].

A different study used quantum mechanics (at the B3LYP/6-311+G* level of theory) to examine the variables that influence the regioselectivity of estrone, equilin, and equilenin metabolism in humans, more precisely, trying to understand the selective oxidation of specific positions in molecules by the CYP enzymes. Thus, they found that increased conjugation (and consequently, electron delocalization) between rings A and B results in an elevation in the nucleophilic character of C4. Such nucleophilic regions (i.e., more electro-rich) are more likely to be oxidized by CYP enzymes, specifically by Compound I, the reactive feryl-oxo intermediate involved in oxidation. The quantum chemical calculations have confirmed that C4 is more susceptible to oxidation than C2 [23].

The B3LYP-631G (p) basis set has also been used to understand the reactivity and stability of uncinatine-A (an acetylcholinesterase inhibitor extracted from *Delphinium uncinatum*), the authors concluding that the compound has strong reactivity and limited stability [24]. Semiempirical methods (MNDO, PM6) have been used to date mostly to characterize the chemical stability and reactivity of natural compounds, such as alternamide (it was concluded it has high reactivity), several compounds isolated from *Coriandrum sativum* L., or menthone and other oxygenated and non-oxygenated compounds from *Mentha longifolia* L. and *Citrus reticulata L*. (Table 1) [25,26,27]. They have also been used to optimize the chemical structures of different natural compounds (e.g., for docking purposes) [28,29,30,31], but they have not been used to directly make inferences or predictions about specific ADME properties.

While hybrid QM/MM methods are being used to some extent for studying the pharmacodynamics of natural products [32,33,34,35] and can also be employed for ADME assessment applications, to the best of our knowledge, such methods have not been applied for this purpose.

### 2.2. Molecular Docking

Since its emergence in the mid-1970s, docking has played a pivotal role in drug discovery and development, as well as in understanding how chemical compounds interact with their molecular targets. Over time, there has been a substantial rise in studies utilizing molecular docking to uncover the structural features required for effective ligand–receptor binding, alongside advancements in refining docking techniques for greater accuracy [36]. Molecular docking has emerged as the method of choice when researchers have access to the three-dimensional crystal structure of their target protein. It has seen widespread adoption thanks to the following two key developments: the dramatic increase in computing power and resources, coupled with the exponential growth of structural databases containing both small molecules and proteins. These factors have made docking both more accessible and more powerful as a research tool [37].

Molecular docking is a modeling technique that examines the interactions between a macromolecule (such as a protein, enzyme, or DNA) and a small molecule (ligand, i.e., the potential drug ingredient). Molecular docking primarily has three objectives as follows: virtual screening, binding affinity calculation, and prediction of the specific spatial position, conformation, and orientation of the ligand [38]. Sampling in molecular docking is challenging due to the vast conformational space, which includes the rotational, translational, and internal flexibility of both the ligand and protein, as well as, in some cases, solvent effects. Exhaustively exploring all possible conformations and orientations within the computational time required for virtual screening is currently unfeasible. As a result, finding efficient sampling methods in docking studies is still an area of active research [37].

Docking studies can be categorized into three types based on molecular flexibility as follows: rigid docking (both molecules fixed), flexible-rigid docking (one molecule flexible, the other fixed), and flexible docking (both molecules flexible) [39]. The first docking methods were based on the idea that molecules have fixed shapes, like a lock and key. This meant that both the receptor (a protein) and the ligand (a drug) were treated as rigid. However, it was later accepted that molecules can change their shapes when they interact (the “induced-fit” model). Because the backbone of a molecule influences the positions of many side chains, allowing both the receptor and ligand to be flexible makes the docking calculation much more complex. However, these flexible docking methods are superior because they more accurately predict not only how molecules fit together but also how strongly they bind to each other [40].

Docking is not mainly regarded as a tool particularly apt for ADME purposes, and there have been literature reviews that have not even considered ADME as a potential application of docking [36,39]. Molecular docking can, however, be used to evaluate the interaction between natural compounds (as well as synthetic or semisynthetic ones) with a variety of proteins involved in different steps of the ADME processes. Thus, a variety of natural compounds have already been evaluated through molecular docking and reviewed for their ability to interact with various transporter proteins relevant for the absorption, excretion or cell efflux [41,42,43].

#### 2.2.1. Docking for Transporter Proteins

A number of publications used docking to investigate the effects of natural compounds on different organic anion and cation transporters. *Cassiae semen* (seeds derived from two species of genus *Senna*) is often consumed as a tea prepared from the roasted product in Korea and China and widely used in Eastern Asia for a variety of medicinal purposes. Wang et al. (2018) have applied molecular docking to predict the interaction between a number of 22 compounds isolated from this product and the organic anion transporters OAT1 (SLC22A6) and OAT3 (SLC22A8). These are located predominantly on the basolateral membrane of the kidney proximal tubules, involved in the initial stage of active renal tubular secretion by uptaking anionic compounds from the blood [44]. For docking, the authors used MOE software (Chemical Computing Group, Montreal, Canada), mostly with the default options; to define the docking area they used SiteFinder, the MOE tool designed to predict theoretical binding pockets on a protein given its structure; and as a scoring function they used London dG. They also used post-docking refinement based on the GBVI/WSA dG method. The authors validated their docking procedure using 10 known inhibitors of OAT1 and OAT3, a step that often tends to be ignored or forgotten when docking is used in natural product research. They reported the binding free energy for all 22 natural compounds evaluated and examined in detail the types of interactions (hydrogen bonds and π–π interactions) that four representative compounds form with OAT1 and OAT3 (in their best docking conformations). The four compounds were aurantio-obtusin (an anthraquinone derivative), 9-dehydroxyleurotinone (a phenolic compound), nor-rubrofusarin-9-dehydrohyroxyeurotinone (a naphthopyrone), and linolenic acid (a fatty acid). The docking predictions of binding free energy matched well with in vitro experimental results (at 100 μM), with compound 3 exhibiting weak binding and no in vitro effects on OAT1 and OAT3, compound 4 exhibiting strong binding and relatively strong inhibition of both transporters, and compound 5 showing selective binding and inhibition of OAT3 over OAT1 [44].

Natural phenolic acids, many with likely beneficial health effects, represent an expanding category of OAT substrates and inhibitors, including caffeic acid, ferulic acid, and gallic acid [45]. Dicaffeoylquinic acids were predominantly excreted in the urine after intravenous administration in rats [46]. These findings suggested that organic anion OATs are involved in the renal handling of these natural phenolic compounds. Using docking, the same research group found that only 3,5-dicaffeoylquinic acid, 3,4-dicaffeoylquinic acid, and 4,5-dicaffeoylquinic acid, but not other dicaffeoylquinic derivatives, interacted with the OAT3 active site, a finding consistent with the in vitro assay results. Moreover, 4,5-dicaffeoylquinic acid formed a stable complex with OAT3 through a variety of intermolecular interactions and exhibited the highest binding affinity among the evaluated phenolic compounds [45].

In addition to in vitro uptake tests, molecular docking was used to examine the selectivity of 10 natural origin inhibitors of the organic cation transporter 1 (OTC1), and benzoylpaeoniflorin was found to be the most selective [47].

P-glycoprotein (P-gp, ABCB1, MDR1, multidrug resistance protein) is an ATP-dependent efflux pump that transports a large variety of structurally diverse hydrophobic and amphipathic compounds. These substances include pharmaceuticals, peptides, and lipid-type molecules. The pump plays a critical role in the efflux of these compounds from cells due to its broad substrate specificity [48]. It has been shown that changes in P-gp activity may impact the oral absorption of medicines, their excretion through the bile or kidneys, and its penetration into the brain. In addition, P-gp is often involved in the multidrug resistance phenomenon of cancer cells [49]. There is no available human P-gp crystal structure available in the RCSB PDB database, but the murine P-gp structure is available and highly similar to the human one. In the various studies performed with natural compounds, certain researchers simply used the mouse P-gp structure, arguing that the sequence similarity is high (around 87% globally and almost 100% within the binding pocket) [50], whereas others, in a more rigorous manner, built homology models to be used in the docking modeling, and some of those have indicated potential differences from the mouse homolog [51,52].

A docking study aimed to investigate in silico the P-gp inhibitory activity of five flavonoids known from in vitro experiments to be inhibitors of this protein (amorphigenin, chrysin, epigallocatechin, formononetin, and rotenone). AutoDock 4.2.6 was used to dock these flavonoids against NBD2 (nucleotide-binding domain 2). The lowest binding free energies (−7.74 to −6.99 kcal/mol) correlated with higher inhibitory activity (R^2^ = 0.7271). Verapamil, a positive control, also showed a relatively low binding energy (−6.97 kcal/mol). Flavonoids exhibited lower binding energies than ATP (−5.27 kcal/mol), indicating competitive binding [53]. The same author group used AutoDock to investigate the binding of 25 flavonoids, along with several control compounds (verapamil, ATP, nifedipine, atorvastatin, and captopril), to the NBD2 domain of P-gp. These flavonoids, representing eight subclasses, included EGCG (epigallocatechin gallate), rotenone, biochanin A, quercetin, phloretin, 5-hydroxy-3,6,7,8,3′,4′-hexamethoxyflavone, silibinin, and naringenin. The authors reported a strong correlation (R^2^ = 0.8941) between predicted binding energies and experimentally observed P-gp inhibition. The following eight compounds were found to have strong inhibitory activity: amorphigenin, epigallocatechin, rotenone, formononetin, chrysin, EGCG, biochanin A, and hesperidin. They exhibited low binding energies (ranging from −7.74 to −6.93 kcal/mol), comparable to the positive control, verapamil (−6.97 kcal/mol). The ability of flavonoids to disrupt ATP hydrolysis and the consecutive inhibition of Pgp was determined by their binding affinity relative to ATP. Given ATP’s binding free energy threshold of −5.27 kcal/mol, flavonoids with significantly lower binding energies were predicted to bind effectively to the NBD2 catalytic site. This prediction was validated by a strong correlation (R^2^ = 0.8941) between the calculated binding energies and the experimentally inhibitory effects of the evaluated flavonoids. This suggests that molecular docking (at the NBD2 site) is a reliable method for predicting flavonoid-mediated P-gp interactions [54].

Neochamaejasmin B is an important active biflavonoid extracted from the dried roots of *Stellera chamaejasme* L. Pan et al. (2015) utilized ligand docking via the FlexiDock module of SYBYL and reported that this biflavonoid exhibits a greater binding affinity for P-glycoprotein compared to (S)-5,7-dihydroxy-2-(4-hydroxyphenyl)chroman-4-one, which was designed in silico by altering the structure of neochamaejasmin B. Their findings suggest that the enhanced binding capacity of biflavonoids to this cellular transporter correlates with an increased presence of hydrophobic features and hydrogen bond acceptors in their molecular structure [55].

The effects of 75 flavonoids on the Pgp have also been evaluated through in vitro and in vivo models, after which the authors have used molecular docking to better understand the nature of the interactions between the five flavonoids that demonstrated significant inhibition and the transporter protein. Digoxin (a recognized substrate of P-gp) was reported to create three hydrogen bonds with tyrosine and alanine residues, while verapamil (used as a positive control) formed Pi interactions with two phenylalanine residues. The five flavonoid inhibitors, all with at least one methoxy group (tangeretin, sinensetin, and isosinensetin—five methoxy groups, sciadopitysin—three methoxy groups, and oroxylin A—one methoxy group) exhibited Pi interactions with Phe974 and Val978 plus van der Waals forces, but these formed no hydrogen bonds with P-gp. Biflavones formed fewer Pi interactions due to a steric hindrance, a fact that could explain the weaker inhibition of sciadopitysin. The latter formed no hydrogen bonds, while amentoflavone (its non-methoxylated homolog) created two strong hydrogen bonds with different binding conformations. Similar results were seen with oroxylin A and wogonin, suggesting that the inhibitory effects of flavonoids depend more on Pi interactions than on hydrogen bonds [50].

Naringenin and dihydrokaempferol were observed to bind in a distinct region from that of the co-crystallized ligand. The authors reported that the interaction energies of the two compounds were comparable to those of rhodamine. However, their estimated binding affinity was slightly lower, and their total estimated energy was slightly higher than those for rhodamine. For naringenin, five hydrogen bonds were identified, whereas dihydrokaempferol formed only one, indicating a relatively weak interaction with Pgp. The authors have used the pdb structure of the murine Pgp, with no discussion of the potential differences in humans [56].

Glabridin, a prenylated isoflavonoid found in licorice roots, has shown various biological effects in non-clinical studies and is known to block P-glycoprotein activity in vitro. The authors optimized its molecular structure using the B3LYP/6-31g(d) level and used AutoDock 4.2 to fit it into P-gp’s binding pocket. Their results indicated that glabridin binds effectively to P-gp, competing with specific substrates like doxorubicin or rhodamine-123 for the drug-binding site [57].

Using docking analysis, Rodríguez-Chávez et al. (2019) examined the way 10 cadinane derivatives from *Heterotheca inuloides* Cass. (Asteraceae) bind to MDR1, MRP1 (human multidrug resistance protein 1), and BCRP (breast cancer resistance protein), revealing that these occur primarily through π-π interactions and hydrogen bonding. The authors optimized the geometries of ligands using the semi-empirical quantum chemistry Austin Model 1 and used AutoDock and AutoDock Vina to carry out the docking. The utilized computational methodology was validated by comparing docking results obtained from co-crystallized ligand data with the docking pose derived from experimental crystal data. Using a Root Mean Square Deviation (RMSD) lower than 2Å as a success threshold, AutoDock and AutoDock Vina yielded very similar results for several compounds (while for others small differences were reported), reinforcing the likelihood that cadinanes can inhibit ABC transporters [58].

To assess the accuracy of molecular docking in predicting P-gp inhibitor affinity, Marques et al. (2021) docked 11 lignans against multiple P-gp structure models. Binding energies and dissociation constants were used as affinity metrics for the top conformations. AutoDock Vina, SMINA, and RF-Score-VS generally failed to produce significant correlations with experimental accumulation data, with only a weak correlation observed for RF-Score-VS with mouse P-gp. While docking predicted strong lignan affinities (values varying from −7.2 to −8.9 kcal/mol with Vina), the predicted Kd values did not entirely agree with the experimental results. NNScore correctly identified the least active lignans but ranked the most potent lignan third. This suggests that docking tools are more reliable for enriching potential inhibitors than at finely separating between compounds with similar activities, especially when activity differences are marginal [51]. Using the same docking protocol, 87 flavonoids were evaluated against P-gp, with AutoDock Vina scores re-evaluated by NNScore, a method previously demonstrated by the authors to be effective for lignans. Many of the screened flavonoids were predicted to have high P-gp affinity, exhibiting nanomolar Kd values. Baicalein and quercetin-3-glucoside displayed the strongest predicted binding affinity. While baicalein’s P-gp inhibition was already established, quercetin-3-glucoside’s interaction was a new finding. However, experimental results contradicted those predictions, showing no accumulation increase in P-gp overexpressing cells. Furthermore, several predicted high-affinity flavonoids showed little to no MDR modulation in accumulation assays. Possible explanations suggested by the experimenters include that fact that P-gp structure optimization for lignans might not be appropriate for flavonoids, different inhibition mechanisms, and limitations related to bioavailability/metabolism limitations [51].

Li et al. (2014) investigated in vitro, in vivo, and in silico the effects of 50 natural compounds of herbal origin P-gp. Four among these herbal compounds (emodin—an anthraquinone derivative, dehydroandrographolide—DAG—a diterpene lactone, 18β-glycyrrhetinic acid—18β-GA, and 20(S)-ginsenoside F1—20(S)-GF1, the latter two having saponin structures) were the strongest inhibitors on Pgp in vitro (on MDR1-MDCKII and Caco-2 cells). Emodin had the highest effect (IC50 = 9.42 μM), followed by 18β-GA (IC50 = 21.78 μM). The other two had substantially lower inhibitory effects on P-gp (IC50 = 76.08 μM for 20(S)-GF1 and 77.80 μM for DAG). The researchers used molecular docking based on the CDOCKER module of Discover Studio to explore binding modes in P-gp’s binding cavity for each compound, and they reported the amino acids interacting with each of the four compounds as follows:

(a) Emodin was involved in four Pi interactions with the Phe974 and Phe728 residues and formed a hydrogen bond through its 3-hydroxyl with Ser975, likely contributing to its strong inhibitory effect.
(b) 18β-GA interacted with Gln191 through two hydrogen bonds, while its isomer, 18α-GA, which had a much lower effect on P-gp in vitro, only had Van der Waals interactions.
(c) DAG formed a hydrogen bond with Gln721, but it was involved in no other remarkable interaction with P-gp.
(d) 20(S)-GF1 formed three hydrogen bonds with Gln721 and Gln191, distinguishing by this from ginsenoside Rh1, which had a very low inhibitory effect on P-gp and binds to Tyr949 via a hydrogen bond [49].

Zeino et al. (2015) screened in vitro 69 natural compounds with a cardiotonic steroid structure and identified a number of six P-gp inhibitors, among which (3β,5β,11α,12α,14β,17β)-3,11-bis(acetyloxy)-12,14-dihydroxybufa-20,22-dienolide (codified by the authors as 15i) exhibited a potency similar to that of verapamil. The researchers explored how the six cardiotonic steroids interact with human P-gp (using a homology model) by conducting molecular docking at two key sites of the protein as follows: the transmembrane domain (TMD) and the nucleotide-binding domain (NBD). The TMD includes three specific drug-binding sites (H, R, and M), while the NBD involves amino acids binding to ATP. Using doxorubicin as a reference, they calculated binding energies, focusing on the lowest values and the most frequent binding conformations. The authors argued that relying on the binding mode that occurs most frequently (i.e., the one with the highest number of conformations) is more likely to reflect the true binding than focusing solely on the lowest binding energy (which is often used as the main criterion in interpreting docking results). This is reasonable, because the lowest energy pose might be an outlier or be less representative of the overall binding behavior of the specific ligand. Their statistical analysis showed a significant preference (*p* < 0.05) of TMD over NBD when considering the most common binding mode; for TMD, the recorded binding affinities were higher (e.g., −8.60 kcal/mol vs. −7.10 kcal/mol for NBD), an observation also confirmed for doxorubicin (−8.06 kcal/mol for TMD versus −7.54 kcal/mol for NBD) [59].

In their 2019 study, Sachs et al. employed rigorous ligand-docking techniques (with careful effort for appropriate validation) to investigate the varying effects of ellagic acid, two isocoumarins (6-methoxymellein and angelicoin B), and nine newly synthesized 3,4-dihydroisocoumarins. Their findings suggest that the differences in the inhibitory effects of those 3,4-dihydroisocoumarins on P-gp-mediated transport, are due to variations in their binding affinities to the transporter, not from their capacity to alter the transport cycle of the protein or otherwise influence its function. These results are in agreement with the proposal that the core cavity of the transporter can hold more than one molecule in distinct subpockets, supporting the idea of a highly adaptable active site of P-gp [60].

*Marsdenia tenacissima* (Roxb.) Wight et Arn contains polyoxypregnanes (POPs), which have exhibited multidrug resistance reversal effects in non-clinical studies. Three POPs were identified as P-gp modulators, capable of restoring the sensitivity of MDR cancer cells to antitumor drugs. To et al. (2017) analyzed the crude extract of *M. tenacissima* and 30 POPs, using flow cytometry to assess the inhibition of P-gp and molecular docking to explore the P-gp interactions with those POPs (the ligand docking was limited to 11 molecules). The most effective POPs were predicted to have strong binding energies (−8.867 to −7.660 kcal/mol), outperforming the P-gp inhibitor verapamil (−7.600 kcal/mol), while an ineffective POP molecule (P10) had weaker binding (−5.677 kcal/mol). P-gp POP inhibitors formed 1–4 hydrogen bonds with key P-gp amino acids (e.g., Leu65, Phe336, Tyr953, and others) in the drug-binding cavity, whereas verapamil was not involved in hydrogen bonding (a finding in line with those reported by [50]). P-gp inhibitors showed a strong preference for the P-gp’s drug-binding domain, located in the space between the alpha and beta subunits [52].

Abraham et al. (2010) investigated how several sipholane triterpenoids isolated from *Callyspongia siphonella* (a Red Sea sponge) interact with P-glycoprotein (P-gp) using molecular docking. For docking, they tested three known P-gp binding sites as follows: those of QZ59-RRR, QZ59-SSS, and verapamil. The docking scores for the QZ59-RRR binding site had the highest correlation with inhibitory activity of the evaluated triterpenoids. Sipholenone E had the highest docking score (6.43), exceeding even the reference ligand QZ59-RRR (6.03), while sipholenol J had the lowest score (4.62). Structurally, rings C and D of sipholenone E align with QZ59-RRR’s isopropyl groups, filling two hydrophobic pockets. Unlike QZ59-RRR, sipholenone E forms a hydrogen bond between its C-10 hydroxyl group and Gln 721, a finding potentially explaining its higher activity. This hydrogen bond was absent in the docking of less active compounds, sipholenols L and J, suggesting that the positioning of rings A and B enhances binding via this H-bond interaction [61]. Although the therapeutic goal in P-gp-related multidrug resistance studies is often pharmacodynamic, the underlying mechanism—transporter inhibition—is inherently pharmacokinetic. Such studies provide essential insights into ADME-related modulation of drug distribution.

The ***breast cancer resistance protein (BCRP, ABCG2)*,** similar to P-gp, is an efflux transporter found in various organs involved in drug pharmacokinetics, such as the gut, liver, kidney, or brain. BCRP can restrict the intestinal uptake of certain drugs, potentially leading to drug–drug interactions when co-administered drugs inhibit intestinal BCRP. Beyond the gut, though, drugs inhibiting P-gp/BCRP typically fail to reach adequate unbound systemic levels at safe clinical doses to block efflux at anatomical barriers like the capillary endothelium of the blood–brain barrier [62]. Previous research indicated that the BCRP structure contains two binding pockets, cavity-1 and cavity-2. The former is considered optimal for the binding of inhibitors [63].

Fan et al. (2019) evaluated the inhibitory effects of 99 flavonoids derived from traditional Chinese medicine (TCM) and foods of plant origin on BCRP. In this context, the researchers used cryo-EM structural data of BCRP for the molecular docking analysis of the most active among them. They used the active site of the internal ligand, refined the protein structure using the CHARMm force field, and docked the flavonoids using the default settings of CDOCKER. They reported that the inhibitory effect of flavonoids is likely related to Pi-Pi stacked interactions and/or Pi-alkyl interactions rather than conventional hydrogen bonds. The spatial docking conformation of flavonoids and KO143 (an inhibitor of BCRP used as a positive control) differed from the substrate mitoxantrone, which forms specific hydrogen bonds and other interactions with BCRP. Mitoxantrone interacts with BCRP through one potential Pi-alkyl bond and two hydrogen bonds, whereas KO143 forms one Pi-Pi stacked interaction and nine alkyl interactions. Inhibitors like KO143 and the 11 flavonoids that demonstrated significant inhibition of the efflux transporter exhibited strong interactions, such as Pi-Pi stacking with Phe439 and Pi-Alkyl with Val546, contributing to their inhibitory strength. The totality of docking data for different flavonoid groups suggested that conventional hydrogen bonds are not crucial for the flavonoid inhibitory effects on BCRP. Hypericin, another important natural BCRP inhibitor, exhibited only Pi-Pi stacked and Pi-Alkyl interactions, reinforcing the idea that these interactions, rather than hydrogen bonds, are key to flavonoid inhibition of BCRP [64].

Banik et al. (2021) used 573 compounds of natural origin from the NPACT database to screen them for BCRP inhibitory properties using a QSAR model developed by the authors. They identified thus 110 compounds, among which only 11 possessed acceptable ADME properties. Nine of them were flavonoid derivatives, one (rohitukine) a chromone alkaloid, and one (7-methoxy-β-carboline-1-propionic acid) a β-carbolin derivative. Using Autodock Vina, the authors explored their interaction with the BCRP protein in cavity 1, and apigenin exhibited a strong binding affinity of −9.0 kcal/mol, consistent with prior studies. It formed π-σ bonds with Met 549 and Val 546, hydrogen bonding with Thr 435, π-π interactions with Phe 439, and various van der Waals interactions with amino acids like Met 549 and Phe 432, in agreement with the known interactions for the ligand MZ29 [63].

Five bisbenzylisoquinoline alkaloids with similar chemical structures (neferine, liensinine, isoliensinine, dauricine, and tetrandrine) have been investigated in vitro for their potential effects on BCRP. Among them, based on cell uptake and transport experiments, liensinine was found a weak substrate of BCRP, while dauricine was an even weaker one. Docking analysis confirmed their interaction with BCRP, showing that liensinine binds to the protein more strongly than dauricine, as indicated by their docking energies (−14.787 kcal/mol for liensinine vs. −8.881 kcal/mol for dauricine). Neferine’s docking energy (−12.955 kcal/mol) was discrepant with the experimental findings, the authors attributing it to software errors, as neferine and dauricine are structural isomers with minimal differences [65].

MRP2 (multidrug resistance-associated protein 2) is a protein that plays a key role in preventing the intestinal absorption of exogenous substances, such as pharmaceuticals. It is primarily found at the apical membrane of enterocytes in the duodenum and jejunum, with decreasing expression towards the distal ileum and minimal expression in the intestinal crypts. It significantly influences the pharmacokinetics and therapeutic efficacy of medicinal products administered orally [66].

Thirty-five structurally diverse flavonoids often encountered in human food sources were evaluated in vitro using MDCK/MRP2 cells overexpressing the protein, and eight of them were found as substrates for MRP2, as follows: two flavones (tangeretin, baicalein), one flavonol (kaempferide), two isoflavones (glycitein, formononetin), two flavanonols (taxifolin, dihydromyricetin), and a flavanolignan (silibinin). Molecular docking calculations using MOE on the drug-binding pocket of a MRP2 model with default parameters indicated that the seven out of the eight flavonoids evaluated (except tangeretin) had low binding energy. A strong correlation (R = 0.926, *p* = 0.003) was reported between the S_scoring and efflux-fold values for the evaluated compounds, suggesting consistency between the in silico docking results and in vitro uptake experiments. The docking results indicated that most flavonoids bind in the transmembrane domain of the MRP protein in a hydrophobic pocket. Methoxyl groups formed hydrogen bonds, highlighting methylation’s role in binding. Those with higher S_scoring values (higher binding energy) had interactions with the protein at more than three sites, whereas those with lower S_scoring values only bound at one or two sites. This suggests that excessive interactions may hinder substrate release after the conformational shift in MRP powered by ATP hydrolysis, and this could explain why flavonoid glycosides with many hydroxyl groups are poor substrates for MRP2, as they bind too strongly to the protein [67].

#### 2.2.2. Docking for Proteins Involved in Drug Distribution

The interactions between natural products and proteins involved in the drug distribution step, such as albumin, α1-acid glycoprotein or hemoglobin can also be investigated in silico using molecular docking. This is how, for instance, the interactions between enantiomers of stipuol (a polyacetylene from *Panax notoginseng* (Burkill) F.H.Chen) and human serum albumin have been evaluated, the authors finding that the natural compounds bind at subdomain III of albumin [68]. It is known that HSA binding affects the actual volume of distribution, and this is essential for understanding its ADME properties [69]. When compounds are free from albumin they tend to distribute and be eliminated faster, whereas when strongly bound to albumin, they tend to circulate less widely and have slower clearance [70]. Thus, such in silico simulations of stipuol’s interaction with albumin offer meaningful insights into its ADME profile.

A study explored cross-docking of bovine serum albumin (BSA) with 7-hydroxycoumarin (7HC, umbelliferone) and 7-hydroxy-4-methylcoumarin (7H4MC, hymecromone). The docking was performed on BSA bound to different ligands (3,5-diiodosalicylic acid, naproxen, and ketoprofen), using Gold 2022.3.0 software (probably with the default parameters, as no details were provided on the procedure), revealing that subdomains IIIA and IB were key binding sites. Experimental data confirmed molecular docking predictions, with naproxen decreasing the binding of both coumarins by ~21%, consistent with docking results indicating perturbations of the interactions between the two natural compounds and BSA by the anti-inflammatory drug [71]. Such docking studies help characterize the extent to which such coumarin derivatives circulate in a bound versus unbound state. Although BSA has certain differences from HSA, such results remain relevant for estimating the systemic PK of natural compounds, particularly in the absence of human clinical data.

Molecular docking was used to analyze the binding of lapachol (a natural quinone derivative present in several tree species from Bignoniaceae), to human serum albumin (HSA), focusing on three possible binding sites identified in drug-displacement experiments. Molecular docking confirmed preferential binding to subdomain IIA (the warfarin-binding site) of HSA, followed by site III (digitoxin-binding site), in agreement with experimental data. Electrostatic potential maps indicated lapachol was more deeply buried in sites I and III than in site II, with hydrogen bonding and hydrophobic interactions contributing to the overall stability of the complex. The ionization state of lapachol at pH 7.4 resembled warfarin, further confirming subdomain IIA (site I) as the primary binding site [72].

Molecular docking indicated that quercetin binds to Sudlow site 1 in the IIA subdomain of human serum albumin (HAS), with a binding energy of −6.46 kcal/mol. Hydrogen bonds and pi-H interactions ensure the stability of the formed complex. A 100 ns simulation confirmed that quercetin remained in place, indicating its strong interaction with HSA and reduced residue fluctuations, and suggesting that quercetin stabilizes the active site upon binding [73]. Mukai et al. (2024), using docking, also highlighted the importance of hydrogen bonds as key interactions between quercetin and HAS, with hydrogen bonds identified between quercetin’s hydroxyl and carbonyl groups and specific amino acids of HSA. However, the amino acid residues identified in the two studies were quite different, except for the Lys195 residue. The latter authors also found that quercetin-7-sulfate binds more strongly than quercetin to site I of the protein. This finding was in agreement with the data derived from the binding characteristics of flavonoids with site-specific HSA-binding fluorescent probes [74]. Unlike these studies that were focused on HSA, Ali et al. (2024) focused on the interaction between quercetin and BSA and reported that quercetin binds to BSA’s active pocket, interacting with Leu115 and Leu122. These amino acid residues establish pi-sigma and pi-alkyl interactions, enhancing the overall binding stability [75]. A study examining the shared binding of amlodipine and quercetin to HSA provides a clear example of a pharmacokinetic drug–drug interaction mediated by competition for albumin binding sites. The authors used molecular docking to complement the wet lab results and found that while occupying essentially the same binding site, amlodipine and quercetin interact with slightly different sets of amino acids as follows: seven of the aminoacids in HAS are common in the two ligands, while three (Glu153, His242, Glu292) are unique to amlodipine, and two (Leu260, Ala291) are unique to quercetin. Also, whereas amlodipine forms a single hydrogen bond with Lys199, quercetin makes two additional such bonds, besides the one with Lys199. These molecular docking results corroborated the spectroscopic measurements performed in the study [76].

Molecular docking was also used to supplement spectroscopic analyses of the interaction between eriocitrin (a flavanone glycoside abundant in lemons and limes, chemically eriodictyol 7-O-rutinoside) and BSA. The results showed that it preferentially binds to subdomain IIA (site I) of BSA, as indicated by the lowest binding free energy. Although hydrogen bonds and van der Waals interactions play a role in binding, the authors concluded that hydrophobic interactions are the most important [77].

Certain natural compounds have been explored for their potential to inhibit HSA glycation in the context of hyperglycemia, because glycated HAS leads to protein remodeling, creation of β-cross-linked structures, conformational alterations, and functional deficiencies in the body [78]. While the primary goal of docking studies on EGCG or colecalciferol has been to assess their ability to inhibit HSA glycation, these investigations also inform ADME-related parameters such as plasma protein binding. Glycation alters the shape and drug-binding characteristics of albumin. Therefore, docking analyses in this area, while primarily focused on pharmacodynamics, also provide valuable insights for predicting pharmacokinetics aspects [79]. Flavonoids with more hydroxyl groups on their aromatic rings have an increased ability to inhibit glycation in HSA [78].

Molecular docking studies evaluating flavonoid interactions with α2-macroglobulin (α2M) provide additional insights into the distribution phase of PK. α2M is an abundant, tetrameric protein that acts as a broad-spectrum proteinase inhibitor, playing a crucial role in the non-specific immune system. Furthermore, it interacts with pharmaceutical compounds, influencing their distribution, excretion, and pharmacokinetic properties. This binding ability makes α2M a significant factor in both endogenous protein regulation and drug disposition within the body [80]. A group of researchers investigated the interactions between several flavonoids (morin [81], myricetin [80], naringenin [82]) and α2M using multiple biochemical and biophysical tools, as well as molecular docking (with Autodock Vina) to better understand the interactions between the protein and ligands and identifying the key amino acids involved in the interaction. Similarly, docking studies of resveratrol identified four possible binding locations where it interacts with α2M. Hydrogen bonds play a key role in the binding process, whereas additional interactions, mostly aromatic in nature, are less significant [83].

#### 2.2.3. Docking for Proteins Involved in Drug Metabolism

The interactions between natural compounds and metabolizing enzymes such as the cytochrome P-450 family of proteins can also be evaluated using molecular docking and a review of this approach for drugs in general (not necessarily natural products) has also been published [84].

Fifteen polyhydroxy-flavonoids were evaluated fluorimetrically for their ability to inhibit CYP3A4. Baicalein, luteolin, and scutellarein exhibited the strongest inhibition among flavones, with IC50 values in the low micromolar range (15–31 μmol/L), while gossypetin, herbacetin, and quercetin were the most potent flavonols, with slightly higher IC50 values (23–40 μmol/L). Molecular docking using AutoDock Vina was used to better understand the interactions between baicalein, herbacetin and luteolin on the one hand and CYP3A4. The top-ranked poses exhibit stronger binding affinities compared to established CYP3A4 inhibitors (cimetidine, diltiazem, fluconazole, and verapamil). They used the ketoconazole to validate the docking model, reporting with a high affinity score (−10.8 kcal/mol) and low RMSD (0.73 Å) against the crystallographic structure. All three flavonoids establish hydrogen bonds with key residues, though the specific residues and functional groups involved differ, all establish π–π stacking and/or π–cation interactions with the heme group, which plays a key role in their binding to CYP3A4. Each compound forms π–alkyl interactions with residues Ala-370, Leu-482, and other nearby residues, and each interacts with multiple additional residues via van der Waals forces, contributing to binding stability [85]. Autodock Vina was also used to explore the interactions of quercetin and six of its glycosides (hyperoside—quercetin-3-O-galactoside, isoquercitrin —quercetin 3-O-glucoside, quercetin-7-O-glucoside, rutin—quercetin-3-O-rutinoside, and quercetin-3-O-sophoroside) with CYP3A4. In vitro data indicated that the glycosylation of quercetin reduced the affinity of the resulting glycosides for CYP3A4. Quercetin exhibited the highest binding capacity to CYP3A4, followed in decreasing order by hyperoside, isoquercitrin, quercetin-7-O-glucoside, rutin, and quercetin-3-O-sophoroside. All six flavonoids docked near the heme group in CYP3A4’s active site and formed hydrogen bonds with surrounding residues, hydrogen bonds and van der Waals interactions being the principal binding interactions. Hyperoside and isoquercitrin had nearly identical docking scores, unlike the experimental results, but otherwise the docking results confirmed the general trend that glycosylation reduces binding affinity (progressively weaker docking energies) [86].

Using DockingServer, Fasinu et al. (2013) showed that verapamil (a known CYP3A4 inhibitor, often used as a reference) and flavonoids naringin and quercetin interact with a hydrophobic cage of CYP3A4 consisting of PHE215, ALA305, ILE369, ALA370, and MET371. They were positioned differently to the ARG212, as follows: the phenyl ring of verapamil was perpendicular, contributing to amine site interactions; the benzopyran ring of naringin aligned parallelly to ARG212, also participating in polar interactions; and the chromenone ring of quercetin was vertically oriented and interacted with ARG212 in a similar manner to verapamil. Verapamil seems to bind more deeply in the hydrophobic pocket, with its amine group interacting strongly with ARG212 and it forms significant cation–π and hydrogen bond interactions, suggesting that it act as a strong CYP3A4 binder. Naringin and quercetin shared similarities but seem to rely more on hydroxyl-driven polar interactions, and this was in agreement with the data of the authors showing that verapamil outperformed naringin and quercetin in terms of CYP3A4 inhibitory ability [87].

Li et al. (2014) used molecular docking to explore the binding of several natural compounds that they found in vitro to inhibit the human CYP3A4 activity as follows: 18β-GA (a saponin), DAG (a diterpene), 20(S)-GF1, and Rh1 (both saponins). 18β-GA and 20(S)-GF1 would inhibit CYP3A4 by strongly binding to Arg212. Emodin, which rather exerted an activating effect on CYP3A4, while weakly interacting with Arg212, strongly binds to Thr310, and this was hypothesized to trigger a change in the shape of the enzyme. Emodin also apparently places itself closer to the enzyme’s heme center compared to midazolam, suggesting it could modify the substrate pocket of the enzyme and enlarge it. However, chrysophanol positions itself closer to the enzyme’s active site, but it is devoid of any activating effect on CYP3A4. The authors have assumed that the lowest docking energy corresponded to the most likely binding conformation, but they neither reported, nor discussed the relevance of the binding energies estimated by docking [49].

Espiritu et al. (2020) conducted molecular docking to identify the preferred orientations of trans-cinnamaldehyde (CA) and 2-methoxycinnamaldehyde (MCA) in the CYP2A6 enzyme’s binding site. Based on the docking results, they used molecular dynamics simulations to evaluate the stability and dynamics of the most important protein–ligand interactions. The researchers developed a structure-based pharmacophore model using crystal structures of CYP2A6-bound inhibitors to guide the docking. All substrates aligned similarly, with one end near the heme’s distal axial coordination site and the other close to the amido nitrogen of the N297 side chain. Most ligands had an H-bond acceptor group and an aromatic ring, likely involved in π-π interactions with phenylalanine residues. The docking, guided by these structural features, positioned CA and MCA with their aldehyde groups near the heme iron and benzene rings close to N297, in line with the expected binding poses [88]. Docking simulations revealed that isoglycycoumarin’s cyclized isoprenyl group is able to penetrate the binding site of the CYP2A6 enzyme, leaving the rest of the molecule distant from 8-methoxsalen (because 8-methoxsalen can block the hydroxylation of isoglycycoumarin, the authors used the co-crystallized structure of 8-methoxsalen with CYP2A6). In contrast, glycycoumarin interacts with the binding site primarily through its benzyl group [89].

CYP2J2 is an enzyme expressed in multiple human cancers and favors tumor cell proliferation by modulating the arachidonic acid metabolism [90]. It is therefore of interest from a pharmacodynamic standpoint but it has also been shown to be involved in the metabolism of certain medicinal products, such as the hydroxylation of ribaroxavan [91]. Docking data using Maestro and a homology model for the protein the authors found that plumbagin (a natural naphthoquinone derivative) binds to CYP2J2 similarly to astemizol, interacting primarily with the enzyme through GLU 222, ALA 223, and ILE 375, the first two residues being already known from previous research as essential for substrate binding [90].

Shukla et al. (2017) docked 10 natural compounds (identified through a more complex screening process) against *Opisthorchis felineus* (the causal ageng of opisthorchiasis) cytochrome P450; this was meant to identify potential inhibitors of this enzyme involved in the resistance of the parasite against other drugs used in the treatment of opisthorchiasis. They used three software products for this purpose (Autodock, Autodock Vina, and Molegro Virtual Docker) and reported that across all three software the interacting residues were similar and that the docking energies showed relatively slight differences [92]. Although this docking study focuses on a parasitic CYP450 isoform, its pharmacological strategy (blocking enzymatic inactivation of antiparasitic drugs) illustrates a pharmacokinetic mechanism (reduced biotransformation) employed to enhance therapeutic exposure at the target site.

Virtual screening models based on machine learning have been used to identify selective CYP1B1 inhibitors (different libraries of compounds, including one of natural products), and the compounds thus identified have then been evaluated for their selectivity against other isoforms by using molecular docking [93]. While aimed at overcoming resistance in cancer therapy, this is in fact a pharmacokinetic strategy. Drug transporters in the kidney are particularly relevant for drug excretion and the interactions of numerous natural compounds with such transporters have been explored through molecular docking [41].

### 2.3. Pharmacophore Modeling

The notion of “pharmacophore” was pioneered by Ehrlich in 1909, who described it as “a molecular framework that carries (Gr. *phoros*) the essential features responsible for a drug’s (Gr. *pharmacon*) biological activity”. Over the past century, the concept has remained, but its scope and significance have been remarkably broadened. Currently (according to IUPAC), a pharmacophore is understood as the complex combination of spatial and electronic features that are essential for optimal interactions with a specific biological target, ultimately initiating or inhibiting a biological response [94]. The pharmacophore model relies on the interaction between ligands and receptors. These interactions encompass all information pertaining to the structural, spatial, and chemical features that underlie specific pharmacological effects. The interaction mostly entails non-covalent bonding, including hydrogen bonding, pi-pi stacking, and ion–dipole interactions, among others [95].

Pharmacophore models are often developed using chemical features found in common between known ligands for a specific target. These are known as ligand-based pharmacophores. Obviously, when only one or several ligands are known for a specific target, or when the available ligands are devoid of structural diversity, ligand-based pharmacophores cannot be used. In such cases, the so-called (protein) structure-based pharmacophore (SBP) models are used [96]. SBP modeling has become increasingly prominent in recent years, driven by the significant growth in high-resolution protein structures. As of January 2025, the Protein Data Bank (PDB) houses approximately 123,000 three-dimensional structures of biological macromolecules, primarily proteins, providing an unprecedented foundation for understanding these molecular therapeutic targets [97]. If experimental structural data for a particular protein are not available, computational methods such as homology modeling can provide an alternative approach to generate a 3D model. Machine learning techniques have also proven effective in predicting protein structures, AlphaFold2 being probably the most widely known example [98].

Developing an effective pharmacophore model demands deep knowledge of the target protein’s structure and binding characteristics. Proteins commonly feature multiple binding sites, and within each site, different ligands can adopt various binding orientations by interacting with distinct regions of the binding pocket. This complexity is further increased by protein subfamily variations, such as those seen among Src kinases, where subtle structural differences between related proteins can significantly impact pharmacophore selectivity [99]. The initial step in structure-based pharmacophore modeling involves selecting and preparing the target protein structure (a step similar in approach and importance for other computational approaches, too, such as molecular docking). This is followed by identifying potential binding sites on the protein. A detailed analysis is then conducted to determine the complementary chemical properties and spatial arrangement of the amino acids at the binding site. Based on this information, pharmacophore features are generated and refined using specialized tools available within the selected software. Finally, the critical pharmacophore features responsible for the biological activity are identified [100]. A detailed description of these steps can be found in the referenced publications and interested readers are encouraged to consult these sources for further information [97,98].

Pharmacophore modeling has been widely applied to predict key ADME properties for natural compounds, particularly for aspects involving transporters and metabolic enzymes.

A pharmacophore model has been recently used in the study of a number of natural compounds (mainly flavonoids) to explore their ability to inhibit organic anion transporter 3 (OAT3) [101], organic cation transporter 1 [102], and organic cation transporter 2 [103]. Although the focus on those transporters was rather related to safety endpoint (prevention of nephrotoxicity or hepatotoxicity), the same approach can be equally relevant for ADME purposes, as biological transporters are involved in ADME processes and drug interactions related to the excretion of certain active substances [104].

Pharmacophore models were also used to evaluate the effects of a number of flavonoids on urate transporter 1 (URAT1) [105]. Based on molecular docking and conformational assessments, the authors built four energy-optimized pharmacophoric (e-pharmacophoric) models using the probenecid-URAT1, benzbromarone-URAT1, lesinurad-URAT1, and verinurad-URAT1 complexes. Through the use of a combination of those models with molecular docking, MM/GBSA (Molecular Mechanics, General Born Surface Area) analysis, and several ADME prediction models, they selected 25 flavonoids from almost 11 000 natural compounds. They evaluated the 25 flavonoids in vitro using a URAT1-overexpressing HEK-293T cell model and found that fisetin, baicalein, and acacetin had the strongest inhibition properties; however, the IC50 values were in the 12.77–57.30 µM range (indicating that they are not very potent inhibitors of this transporter) [105].

Pharmacophore models have also been used to explore the effects of certain natural products on specific CYP450 fractions, such as those of saponins [106] or flavonoids [107] on CYP3A4 or those of a variety of natural compounds on CYP1A1 [108,109]. Thus, because previous research on ginseng extracts and ginsenosides’ effects on P450 enzymes seem to have yielded conflicting results, a recent study was performed, where the researchers examined 15 ginsenosides and sapogenins’ impact on five major human drug-metabolizing P450 enzymes in wet experiments. They then employed the HipHop algorithm (available as part of the Catalyst software, v. 4.10 (Accelrys corporation, San Diego, CA, USA) to develop a pharmacophore model; this comprised one hydrogen-bond acceptor, one hydrophobic point, and two hydrogen-bond donors. Hydroxyl groups at positions C3 and C12 were found to be essential for activity, and substituting these groups with other moieties reduced the potency of those compounds [106]. HipHop was also used to build a pharmacophore model for P-gp inhibition by flavonoids. The training set consisted of only six flavonoids, of which three were potent inhibitors, two moderate inhibitors, and one had no inhibitory activity. To validate the model, they mapped 20 flavonoid molecules onto it. The pharmacophore model suggested that flavonoids with Pgp inhibitory properties share an aromatic ring, a number of hydrogen bond acceptors, and the presence of certain hydrophobic groups. For instance, adding hydrophobic groups at the 6- and 7-positions of the B benzene ring significantly increases their ability to inhibit P-gp. Isosinensetin with these hydrophobic groups showed stronger inhibition than oroxylin A, which mostly lacks them. Similarly, sciadopitysin (with three methoxy groups) was more potent than amentoflavone and wogonin (both more hydrophylic) [50]. A similar approach was used by the same research group to identify which structural characteristics were essential for activating CYP3A4. The authors found that CYP3A4 activators typically possess the following three key features: a B aromatic ring structure, hydrophobic moieties at position 7, and hydrogen bond acceptors at position 4. Molecular docking experiments provided supporting evidence for these structural requirements [107].

Tahir et al. (2019) used molecular docking and pharmacophore modeling to study compounds that interact with CYP1A1, including natural products like alkaloids, coumarins, flavonoids, and others. They generated 10 pharmacophore models and screened them against two ZINC sub-libraries (one of which was based on natural compounds), selecting the 50 highest-scoring compounds. They also built an additional model using the most potent compound from each class. Their key findings were that bergamottin (a coumarin) had the highest Gold fitness score, and a different coumarin, ZINC08792486, exhibited the lowest binding energy in docking [108].

Hochleitner et al. (2017) re-used a pharmacophore model developed over a decade prior to screen for natural compounds as inhibitors of CYP2D6. Their in silico screening of 2147 plant-based compounds yielded 75 hits satisfying the model’s features. Among these, 23 compounds were subjected to experimental validation and 10 demonstrated strong inhibitory activity and 8 moderate activity at 100 µM concentrations. Chelidonine was identified as the most effective inhibitor of CYP2D6 in this study [110].

### 2.4. Quantitative Structure–Activity Relationship (QSAR) Models

QSAR modeling is a computer-based method that establishes empirical correlations between chemical descriptor values derived from molecular structures and experimentally obtained properties or bioactivities. These models are then used to predict or design new chemicals with specific properties [111]. In other words, the chemical structure of a substance can be described by a series of numeric features named generically “molecular descriptors” (e.g., molecular weight, number of double bonds, percentage of oxygen atoms, and others much more sophisticated) and QSAR establishes mathematical relationships between these numeric values and certain biological activities of interest (for instance, the inhibition or not of a certain CYP450 isoform, the percentage of oral bioavailability, etc.).

The use of QSAR has undergone significant changes in recent decades, and these include the following: (1) using more detailed descriptions of molecules, from simple one-dimensional (1D) representations to more complex multi-dimensional (nD) ones; and (2) employing more sophisticated methods, including machine learning, to correlate molecular structure with biological properties. Initially, QSAR studies were limited to small groups of closely related chemicals and relied on basic regression analysis. Now, QSAR can handle much larger and more diverse datasets, using a wide range of machine learning techniques for both modeling and virtual screening [112].

Based on the assumed relationship between the chemical descriptors and the biological effect modeled, QSAR models can be classified into linear (e.g., based on multiple linear regression or partial least squares) and non-linear (e.g., support vector machines or artificial neural networks) [113,114].

Based on the mode in which descriptors are computed and models are built, QSAR models with different dimensions are distinguished as follows: 2D-QSAR, 3D-QSAR, and more recently 4D-, 5D-, 6D-QSAR models have been proposed. The 2D-QSAR models are based on descriptors computed based on the 2D representation of ligand structures (but ignoring completely the 3D shape and spatial arrangement of atoms within the ligand) [115]. 3D-QSAR correlates biological target properties with molecular descriptors derived from 3D chemical structures; it uses probe-based sampling within a molecular lattice to compute the 3D molecular descriptors [116]. In a paper published in 1997, Hopfinger et al. proposed for the first time the concept of 4D-QSAR [117]. The fourth dimension, known as ‘ensemble sampling’, consists of a set of molecular configurations, describing ligands in different molecular and spatial forms, such as conformations, orientations, stereoisomers, and protonation states [115,118]. First proposed by Vedani and Dobler in 2002 [119], the 5D-QSAR approach addresses the induced-fit phenomenon in the ligand–protein interactions by considering multiple conformations that a ligand can adopt when binding to its target. This flavor of QSAR, which builds upon 4D-QSAR, creates virtual representations of these different binding scenarios to improve the predictivity of the model(s) [120]. 6D-QSAR builds upon previous QSAR versions by adding a sixth dimension—solvation. It allows the simultaneous evaluation of multiple solvation models to better understand how solvents influence molecular interactions. This can be carried out explicitly by mapping solvent properties onto the molecular surface or implicitly by adjusting the contribution of solvation terms (like ligand desolvation and solvent elimination) for different models within the analysis. During the simulation, weights associated with different conformations are adjusted through an evolution process [121].

Mechanistic QSAR is an approach that employs parameters/descriptors that have a consistent and unambiguous meaning, aiming to provide insights into the underlying mechanisms of the predicted biological effects. The alternative, named sometimes non-mechanistic QSAR, prioritizes the development of equations with predictive value, even if the parameters used are not readily interpretable. This approach focuses on statistical correlations between molecular descriptors and biological activity, but it does not necessarily offer mechanistic insights into the relationship between the descriptors and the biological effects (e.g., Comparative Molecular Field Analysis—CoMFA) [122,123].

A global QSAR model has been defined as a model characterized by a data set that is highly structurally diverse, which in turn reflects a variety of mechanistic actions among the chemical compounds of the data set. In such a model statistical methods are employed to attempt to identify structure/activity patterns that are independent of the mechanistic differences [124]. It has been stated that despite the fact that statistics might indicate that these models are robust, they tend to exhibit relatively low predictivity. Local models, of the contrary, are characterized by structural or mechanistic similarity [124]. The heterogenous definition of global QSAR models is somewhat problematic, and it could gain clarity by distinguishing between structurally global models (models built on a variety of structurally diverse compounds) and mechanistically global models (models built on chemical compounds acting through a variety of mechanisms and on distinct biological targets). There are theoretical reasons to believe that the latter have poorer performances than the former.

Sometimes QSAR models are qualified by the nature of the chemical descriptors used in their construction, for instance “topological QSAR” models (based on topological descriptors) [125,126] or “quantum mechanical QSAR” models (based on quantum mechanical descriptors) [127]. The “hologram QSAR” technique involves the decomposition of each molecule in the data set into a molecular hologram. This hologram is primarily composed of linear, branched, and overlapping fragments, which are then arranged in an array of fixed length (ranging from 53 to 401 bins). These bin values serve as X variables in QSAR modeling, capturing both the composition and topology of molecules. Several factors affect how the hologram is generated and how the resulting HQSAR models perform statistically, including the following: the length of the hologram, the size of fragments, and how fragments are distinguished (based on atom types, bonds, connections, hydrogen atoms, chirality, and groups of hydrogen bond donors or acceptors) [128].

QSAR models can also be classified based on the categorical or continuous nature of the predicted outcome, distinguishing between classification and regression models. Classification models use chemical information of the training data set to make predictions about an outcome category, most often using a binary category (e.g., active/inactive, orally bioavailable/non-bioavailable, etc.). Often such a classification involves an arbitrary conversion of a continuous variable in two categories at a certain threshold. It is argued that in such a case, “the predictive reliability of the resultant QSAR model will increase at the expense of the resolution of the prediction” [129]. Regression QSAR models make predictions about the outcome variable on a continuous scale (predicting, for instance, a numerical value of ki, IC50, or of a percentage for the oral bioavailability and not a mere category) [130].

Biological effects that can be predicted using QSAR models can be vastly diverse, from the effects on a specific biological target to the prediction of a specific adverse effect or a specific ADME feature. Among the in silico methods for ADME predictions, QSAR models have been probably used the most extensively, and generally, the same models can be applied for both natural and synthetic organic chemical compounds, although often the specific use of such models for natural compounds has not been explicitly mentioned [131,132].

Today, tools such as ADMETlab 3.0, SwissADME, and others (discussed below in Section 5), which are machine learning-driven QSAR/QSPR applications, offer reliable predictive accuracy for natural compounds. This is due to their training on large datasets and the utilization of a variety of chemical descriptors.

Kansy et al. introduced the parallel artificial membrane permeation assay (PAMPA) in 1998 [131]. PAMPA is composed of lipophilic filters that are coated with lecithin in an organic solvent solution. Transcellular permeation is assessed by a rapid in vitro assay system that is suitable for high-throughput screening. Akamatsu et al. (2009) constructed an in silico prediction model of human oral absorption for potentially transported compounds by developing a QSAR model that describes correlations between chemical structure and PAMPA permeability [132]. This study has not mentioned explicitly the use of the QSAR models for natural compounds, but similar QSAR models based on PAMPA have been developed and one has been used to predict the gastro-intestinal absorption of flavonoids from *Silybum marianum* (L.) Gaertn [133,134].

Oral clearance refers to the rate at which a substance/drug ingredient is removed from the body following oral administration. Boik and Newman (2008) developed three QSAR classification models based on data from human oral clearance, rodent LD50, and in vitro cytotoxicity studies. Then they used these models to analyze over 115,000 natural compounds, resulting in the prediction that hundreds of these substances exhibit low systemic toxicity, low to moderate oral clearance, and manageable cytotoxicity levels [135]. There are numerous other QSAR/QSPR models that have been developed to predict the oral absorption of drugs, although they have not necessarily been applied to natural compound datasets (Table 2).

Fang et al. (2018) developed a 2D-QSAR model based on the Caco-2 uptake of 21 flavonoids (used as the training set) using molecular properties and topological descriptors (computed with Sybyl X-2.0 and MOE). Their selected model used two descriptors—QC5 (the partial charge on the carbon atom at position 5) and SlogP (a BUCT descriptor whose higher values indicate higher hydrophobicity). The cellular uptake (CU) of flavonoids tended to increase with greater hydrophobicity, as indicated by SlogP values. Flavonoids with higher SlogP values had higher CU and those with lower SlogP had lower CU but optimal CU was found for moderately high SlogP values [159].

Gonzales et al. (2015) constructed both 2D- and 3D-QSAR models to understand and predict how well flavonoids are absorbed in the intestine, based on results of Caco-2 cell model results. Stepwise multiple linear regression (SMLR) and partial least squares regression (PLSR) 2D models showed reasonably high external predictive capabilities (Q^2^ values of 0.77 and 0.67, respectively). Because the descriptors on which those models were based were not easily interpretable, they developed a COMSIA model, with a slightly inferior (0.63) Q^2^. The latter model was based exclusively on hydrogen bond acceptors and donors as key descriptors, as including hydrophobicity did not enhance predictive accuracy, while steric and electrostatic factors had no significant impact on flavonoid intestinal permeability. This model indicated that flavonoid glycosides, particularly those with a glucose unit at the C3 position, exhibit poor transport across Caco-2 cells due to their hydrogen-bonding properties [160,161].

Wu et al. (2011) built two 3D-QSAR models for UGT1A9 (one of nine UGT1A isoforms known for their key involvement in glucuronidation of flavonols at the C3–OH position), using Vmax and CLint as dependent variables. Their models (trained on 23 flavonols and evaluated on seven additional ones), with good external predictability (R^2^pred values of 0.74, and 0.63), were developed after aligning training molecules using a pharmacophore model that featured the glucuronidation site at the C3–OH position and the two aromatic rings of the flavone nucleus. Their models indicated that the hydroxyl groups in both rings significantly enhanced glucuronidation susceptibility to the C3 hydroxyl. Additionally, bulky substituents (such as methyl groups) near positions C5 and C6 also tend to increase the likelihood of glucuronidation [161,162]. The same research group then developed COMFA and COMSIA models for a wider variety of natural phenolics (including a large number of flavonoids but also other structurally diverse phenolics, including curcumin and emodin, for instance), using a total data set of 145 molecules (both training and test sets). They reported good performance for their models (r^2^pred 0.78 and 0.70) but critics doubted their usefulness, arguing that aligning structurally diverse molecules impacts the reliability of the 3D models, “considering that molecular alignment is a crucial step in COMFA and COMSIA” [163].

Wang et al. (2005) developed a 2D-QSAR model on 57 flavonoids’ data compiled from the literature, using a Bayesian-regularized neural network. The models were meant to predict their P-gp inhibitory properties and the outcome data were based on their binding to the nucleotide-binding domain located at the C-terminal end of mouse P-gp [164]. Similarly, Sheu et al. (2010) used 22 flavonoids to build a 2D-QSAR model predicting their effects on P-gp, using outcome data obtained experimentally on HCT15 cells. Their best model indicated that the presence of hydroxyl groups at C3′ and C4′ hinders the ability of flavonoids to inhibit P-gp, whereas at hydroxyl groups at C7 and C5′ favors the P-gp inhibitory effect; also, flavanols tend to inhibit P-gp, whereas isoflavones do not. ClogP has a slight favorable effect on the P-gp inhibitory activity. Unfortunately, the model was developed on a very small data set and no validation was reported by the authors [161,165]. 3D-QSAR models (CoMFA and CoMSIA) were developed by Kothandan et al. (2011) for similar purposes, using a data set of 41 flavonoids (32 used for training purposes and 9 for testing). The models had good performance and indicated that introducing bulky, non-polar groups at the 6th and 8th positions on the A ring is favorable for the P-gp inhibitory activity; instead, electrostatic interactions and hydrogen bond capabilities seem negligible in their influence on the P-gp activity [166].

A QSAR model describing the MRP1 inhibition by flavonoids was developed by van Zanden (2005) using a data set of 29 compounds. Their best model was developed through linear regression using three descriptors and it indicated that the inhibitory activity was favored by increasing the number of OH or OCH3 groups, and it was negatively impacted by a higher dihedral angle between the B and C rings [167]. However, the authors did not report on any validation attempt and the model validity was doubted in the literature because an increase in the number of OCH3 moieties on the same structure should tend to decrease the number of hydroxyl groups on the same structure [161]; the resulting model configuration, though, is probably due to the relatively small size of the training set.

For the BCRP transporter, Zhang et al. (2005) developed a model on a data set of 25 flavonoids (19 used for training purposes and six for testing) and three descriptors selected with a genetic algorithm out of 115. According to the model (validated using LOO with a 0.78 Q^2^), the inhibition of BCRP is favored by the computed octanol–water partition coefficient (logP) and the count of all atoms with a double bond, and it is disfavored by the “moment of the displacement between the center-of-mass and the center-of-dipole along the inertial *Y*-axis”; the authors acknowledged that those descriptors (particularly the latter) are not easily interpretable (are not intuitive for the human mind) [168]. Nicolle et al. (2009) applied a 3D linear solvation energy analysis combining MIFs (molecular interaction field) and VolSurf descriptors to study new flavonoid inhibitors of BCRP (both natural and synthetic). In contrast to other 3D-QSAR techniques that utilize MIF mapping, such as CoMFA and CoMSIA, VolSurf models do not require the alignment of molecular structures. This is because VolSurf condenses the spatial distribution and intensity of molecular interactions encoded by each MIF into one-dimensional descriptors, eliminating the dependency on molecular alignment. Their model, based on 34 compounds, accurately predicted inhibitor classes (r^2^ = 0.77, q^2^ = 0.70) using descriptors like DRY polarizability, hydrophobic volumes, and shape parameters (volume, surface, rugosity, and globularity). Model reliability was confirmed with seven external flavonoid derivatives, showing good correlation (r^2^ = 0.67–0.71, Spearman = 0.75–0.80) between the predicted and measured inhibitory activity against BCRP in HEK-293 cells [169].

Another study investigated the inhibitory effects of various flavonoids on BCRP using both 2D- and 3D-QSAR models. For the QSAR modeling, only the active flavanones and flavones were included, while inactive compounds, biflavonoids, and genistein were excluded. The 3D-QSAR analyses employed CoMFA and CoMSIA to identify molecular fields influencing inhibitory potency. In CoMFA, the hydrogen bonding field used alone produced the best model, whereas the addition of steric or electrostatic fields slightly reduced the cross-validated coefficient (q^2^). For CoMSIA, the optimal model combined electrostatic and hydrogen bond acceptor fields, achieving a q^2^ of 0.624. The authors also validated the models’ robustness using a scrambling stability test. Further validation using an external data set demonstrated a correlation coefficient (r^2^) of 0.63, confirming the models’ good predictive capabilities. The analysis also revealed two distinct clusters corresponding to flavanones and flavones, suggesting differences in structural influences on BCRP inhibition. The COMSIA model indicated that hydrogen bond acceptors near C5–C7 enhance BCRP inhibition, suggesting that hydroxyl/methoxyl groups in these regions tend to elevate BCRP inhibition. Conversely, a C3–OH group diminishes inhibition, while a negative charge at C3 and positive charges at C2/C4 improve BCRP inhibition—highlighting the role of polarizability. These findings explain why flavones exhibit stronger BCRP inhibition than flavanones, and thus the two clusters identified by the authors [161,170].

QSAR models have also been developed to predict plasma protein binding [171,172,173,174] or the unbound fraction [175]; the volume of distribution in humans, rodents, or other mammals [176,177,178]; the blood–brain barrier (BBB) permeability [179,180,181]; inhibition of specific CYP450 fractions [182,183,184,185]; elimination half-life [186], rodent [187,188], and human clearance [189] effect of substances on key transporters such as P-glycoprotein [190,191,192]; organic anion transporters [193,194,195]; organic cation transporters [196]; etc. Some of the models referenced here have been specifically developed for natural products (for instance, most of the published models have been developed for medicines or organic substances irrespective of their natural or synthetic origin.

Li et al. (2018) explored the quantitative relationship between the structural characteristics of flavonoids and their ability to inhibit CYP3A4. Their data set consisted of 44 flavonoids (32 used for training and 11 for testing) and they used a 3D-QSAR model using CoMFA descriptors and the partial least squares (PLS) method. Their model indicated that flavonoids with bulky groups in the A ring or hydroxyl groups at the R-3 and R-2 positions, as well as electropositive groups at the R2-4 position favor inhibitory activity against CYP3A. Instead, ring-closure at the R2-4 position significantly tends to cancel the inhibitory effect [184]. Vázquez et al. (2014) built multiple 2D-QSAR models to predict the affinity of flavonoids to the P-glycoprotein transporter, using a data set of 62 flavonoid molecules (with the four prototype structures: flavone, chalcone, flavanone, and isoflavone). Their findings indicate that hydrophobic and geometric factors are crucial for binding, with electronic factors also playing a role in the derivatives containing flavone, flavonols, flavanone, and isoflavone cores, through electron donor/acceptor interactions. For chalcones, the binding to P-gp is mainly driven by dispersive forces within the binding pocket [197].

Although most QSAR studies focused on PK properties of natural compounds have focused on flavonoids up to date, such models have also been developed and applied to predict ADME properties of alkaloids [198], terpenes and terpenoids [199], or saponins [200].

Certain molecular descriptors can be computed/estimated using molecular dynamic simulations, a case in which QSAR and MD simulations are integrated in a single final model. For instance, Iyer et al. (2007) have used MD to simulate the behavior of different ligands across a dimyristoylphosphatidylcholine (DMPC) monolayer membrane and compute a number of descriptors used then to build QSAR models predicting the intestinal oral absorption of the drugs included in the models. The test and train compound datasets included both natural and synthetic substances [146]. Other relevant descriptors can be computed using the DFT theory, a case in which QSAR and DFT are integrated in a single model (see, for instance, ref. [144]). Another hybrid approach has been recently (year 2024) reported for 48 compounds, mostly natural (including alkaloids, flavonoids, and coumarins). The authors built a QSPR model to predict the apparent permeability coefficient (Papp) (measured on Caco-2 cells), and then used MD simulation to evaluate the molecular mechanisms involved in the absorption differences observed for ligustrazine and EGCG, as well as molecular docking to understand the interactions between the compounds and the P-glycoprotein [201].

### 2.5. Molecular Dynamics (MD) Simulations

X-ray crystallography provides static snapshots of molecules because they are locked in a crystal. This led to an initial perception that large molecules like enzymes and receptors were rigid. However, combining multiple snapshots often reveals that these molecules exist in different states, indicating they are actually dynamic and fluctuate between these states [202]. Molecular dynamics simulations use physics-based modeling to predict how individual atoms move within proteins and other molecular systems over time. These highly precise simulations can track atomic positions down to the femtosecond level, allowing researchers to acquire knowledge about important biological processes like protein folding and ligand binding. The simulations provide valuable insights into how biomolecules respond at the atomic scale to various changes like mutations or chemical modifications. This computational approach has become a powerful tool for understanding molecular behavior and interactions [203]. The method was for the first time applied to proteins at the end of the 1970s [204]. The applications of MD simulations in the fields of molecular biology and drug discovery are vast, but we are here focused on their potential use in making ADME predictions. It seems likely that the most important such applications consist in improving molecular docking results for the interactions between different ligands and the proteins of interest, relevant for pharmacokinetics purposes.

#### 2.5.1. Applications for Natural Products

Only a few applications of MD simulation in predicting ADME properties of natural products have been published to date. All ligand–protein interactions that can be studied for ADME purposes using molecular docking can also be better understood using MD simulations, and the applications of the former are also applications of the latter. But MD simulations have also been used for certain processes relevant for ADME but less investigated with molecular docking. The most important such application is the study of membrane permeability. In 1994, Marrink and Berendsen performed the first atomistic simulation of water permeability in a lipid bilayer. Since then, hundreds of coarse-grained and atomistic simulations have been carried out to study the passive transport of permeants across different lipid bilayers and synthetic membranes [205]. Molecular simulations are useful for studying both synthetic and natural substances. Two studies specifically investigated how menthol enhances the skin’s ability to absorb substances [206,207]. These studies showed that menthol readily penetrates the skin’s protective outer layer (lipid bilayer), making its interior more fluid. However, at high concentrations, menthol can damage this layer. Borneol, another natural compound, at low concentrations helps substances penetrate the skin, but at high concentrations creates pores and unusual structures called reverse micelles, a mechanism similar to how some antimicrobial peptides disrupt cell membranes (the “carpet mechanism”) [205,208,209]. MD simulations are widely used to study drug-membrane systems, revealing molecular-level interactions and thermodynamic properties of drug products (both synthetic and natural) that align well with experimental results. Such MD simulations for natural products have covered several classes of compounds and they have been reviewed elsewhere [210], but generally, the purpose of those investigations has not been of a PK nature.

Ibrahim et al. (2023) simulated the interaction between natural compounds and the ABCB1 transporter within a 1-palmitoyl-2-oleoyl-phosphatidylcholine (POPC) lipid bilayer membrane, the authors centering the ABCB1-inhibitor complexes within the POPC membrane structure. Computational modeling was performed using the Lipid14 force field parameters through the AMBER16 software package. They reported that the presence of the POPC bilayer had no notable effect on the binding energies of the natural compounds within the ABCB1 binding pocket, as compared to the results obtained without POPC [211].

Molecular Mechanics—Generalized Born Surface Area (MM-GBSA) was used by Ibrahim et al. (2022) to assess the interaction of several hundreds of natural compounds with ABCG2 (ATP-binding cassette transporter G2), an efflux transporter contributing to multidrug resistance. The most promising compounds found (with estimated free energy below −60.5 kcal/mol) were complexed with the ABCG2 transporter and then put through MD simulations. To increase efficiency, the authors used an implicit water solvent and a relatively brief duration of 250 ps. Longer MD simulations (1 ns) in implicit solvent followed by MM-GBSA calculations were performed for 238 complexes of natural compounds with the ABCG2 transporter, identifying 21 compounds with stronger binding affinities than a reference inhibitor (BWQ). Those 21 compounds were further analyzed using 25 ns MD simulations in explicit solvent, and thus two compounds were found with strong binding to ABCG2. For those two compounds (tannic acid and 1,2-Di-O-(9Z,12Z,15Z-octadecatrienoyl)-3-O-(6-p-hydroxy-phenyl-propionamido-6-deoxy-alpha-D-glucosyl)-glycerol) MD simulations were extended to 100 ns and binding energies were subsequently calculated. In their 25 ns and 100 ns simulations, the authors found the binding affinity evaluated with MM-GBSA for the two natural compounds associated with the ABCG2 transporter to be essentially the same. Through this approach they identified two prospective ABCG2 inhibitors to be used for further development [212].

#### 2.5.2. Refinement and Integration with Other Methods

While molecular docking can predict how a protein and a ligand interact, the initial prediction of how the ligand is positioned and oriented within the protein’s binding site is often approximate. To obtain a more accurate picture of the interaction, further refinement is necessary. MD simulations can refine the docking results by minimizing steric clashes (where atoms are too close together) and adjusting the initial binding position of the ligand [213]. For instance, Sabiu and Idowu (2021) have found using ligand docking on CYP3A4 and found that glycyrrhizin had the best docking score (−9.1 kcal/mol) among the four compounds evaluated, and myricetin had a lower docking score (−8.1 kcal/mol). They used MD simulation to obtain additional insights into the binding affinities and structural dynamics of the interactions, and this time, they found that myricetin had higher binding energy and stable conformational dynamics in molecular simulations. The in vitro results were more in line with the MD simulation; myricetin demonstrated a concentration-dependent inhibitory effect on CYP3A4 activity with an IC50 value of 10.5 ± 0.55 μM, whereas glycyrrhizin exhibited no inhibitory effect on CYP3A4 activity in vitro, despite its strong in silico binding affinity. RMSF (root means square fluctuation) quantifies how the binding of a ligand affects the properties of the active site residues in a protein. In this case, the RMSF value for the alpha carbon fluctuation was similar for myricetin and ketoconazole, indicating a higher likelihood of CYP3A4 inhibition for myricetin than for glycyrrhizin [214].

To enhance the effectiveness of virtual screening, MD simulations can be combined with binding free energy calculations to evaluate the binding energies of small molecules with a target (thus understanding how strongly a ligand binds to the target of interest). These energies can then be used to rank candidates and refine the scoring of generated poses. Methods like Molecular Mechanics Poisson–Boltzmann Surface Area (MM-PBSA) and Molecular Mechanics Generalized Born Surface Area (MM-GBSA) enable more precise calculations of the binding free energy between the ligand and receptor. Entropy changes are estimated through normal mode analysis, while enthalpy changes for the entire system are statistically derived from electrostatic, van der Waals, and solvation energies, all based on MD simulation trajectories. This approach allows for the accurate determination of binding free energies [213]. Whereas MM-PBSA and MM-GBSA are not themselves MD simulations, they use the MD-generated data to estimate more or less accurately the free energy and thus to better understand the ligand–target interaction.

### 2.6. Physiologically Based Pharmacokinetics (PBPK) Modeling

While PBPK modeling has been widely used to predict the pharmacokinetics of synthetic drugs [215], with no less than 74 cases examined by the FDA in the last 5 years, its application to natural products is now on an ascending track. Such models not only facilitate dose optimization and tissue distribution predictions for natural products, but can also be very useful in evaluating herb-herb or herb–drug interactions, a key consideration in the therapeutic use of natural products, allowing the clinicians to better evaluate the interaction risk, to personalize the posology, and to ensure therapeutic success [216].

PBPK models simulate how a drug and its metabolites change in concentration over time in the blood and target organs. Building PBPK models, however, requires more effort and data compared to simpler predictive methods. They necessitate estimating a larger number of parameters and demand extensive knowledge of the body’s physiology and the specific drug’s properties [217]. The model’s parameters are obtained either from non-clinical experimental data, clinical trials or calculated based on appropriately validated formulas. Broadly, these parameters can be categorized into two distinct groups as follows: drug-specific parameters and organism-specific parameters. Although a PBPK model is usually very complex, with hundreds of ordinary differential equations, a large number of parameters, particularly organism-specific ones, are already known (e.g., organ blood flows, tissue volumes, membrane permeabilities, etc.); therefore, for a new medicine, often less than five drug-parameters are needed (e.g., clearance, volume of distribution, binding parameters, etc.) [218].

PBPK models estimate drug kinetics in one or several organs, necessitating a submodel for each organ or tissue considered and then integrating the organ submodels into a whole-body, comprehensive model. Drug transport occurs via blood circulation, as determined by anatomical structures. PBPK strives to replicate authentic, quantifiable physiological and/or pharmacokinetic processes instead of more abstract rate constants and volumes. Parameters used in PBPK models may be ‘intrinsic’ covariates, such as weight, body surface area, or glomerular filtration rate, rather than covariates identified post hoc to improve model fit (such as, for instance, the patient age, in the absence of a clear mechanism or explanation of why it would improve fit) [219]. A PBPK model for a specific medicine can be validated using both in vitro data and in vivo oral pharmacokinetic data from rats. To predict the human oral pharmacokinetic profile, the physiological and in vitro parameters from the rat model can be substituted with human-specific parameters, following a successful prediction of the rat’s in vivo oral pharmacokinetics from in vitro data [220].

Compartmental models, widely used in the traditional pharmacokinetic research, are built using a “top-down” method, where all the model’s information is derived from a specific set of pharmacokinetic and covariate data. Conversely, PBPK models tend to use a “bottom-up” approach, where the model’s information is derived from in vitro data and pre-existing knowledge of physiological and pharmacological mechanisms involved in the drug pathways through the body, the model parameters being thus derived from mechanistic principles (the focus is not so much on merely fitting the data as to providing a mechanistic understanding of the drug behavior) [219].

PBPK models offer valuable predictions for various drug development purposes as follows:

- At the clinical trial design stage, they can predict how drug formulation and food intake will influence pharmacokinetics, guiding initial human studies and forecast drug–drug interactions mediated by enzymes or transporters, informing inclusion/exclusion criteria, dose selection, and potentially waiving unnecessary clinical interaction studies or studies where enrolling subjects is anticipated to be difficult.
- They can predict appropriate dosing regimens for different pediatric subsets, from newborns to adolescents, by enabling informed selection of sampling timepoints and proposing suitable doses.
- They can predict exposure to the drug in patients with impaired renal or hepatic function, guiding organ impairment studies or supporting decisions to waive such studies.
- They can estimate the drug disposition in the mother and fetus, aiding in optimizing the therapeutic benefit-risk ratio during pregnancy.
- They can predict pH-mediated drug–drug interactions in patients receiving proton pump inhibitors or antacids, guiding formulation development and efforts to minimize food–drug interactions [221].

It is not feasible to cover technical details on building PBPK within the limited space of this paper, but they are comprehensively addressed in the literature, with several high-quality tutorials providing valuable guidance on different aspects of the modeling process [218,222,223].

A limited number of studies have used PBPK modeling up to date to approach ADME properties for natural products. For instance, a recent study reported its use to estimate the biodistribution of *Centella asiatica* administered orally in a rat model [224]. Another study used a PBPK model to simulate the drug–drug interaction between aconitine and the glycyrrhizic acid, using parameters derived from in vitro experiments. The model simulations were validated against experimental observations to evaluate their accuracy. The analysis focused on understanding how CYP3A and P-glycoprotein contribute to the reduction in aconitine toxicity induced by the glycyrrhizic acid [225]. Similarly, PBPK models were used to explore the interactions between silybin A and losartan [226], between schisandrin A, schisantherin A and cyclophosphamide [227], the drug–drug interactions involving bergamottin (from grapefruit juice), curcumin (from turmeric), and hyperforin (from St. John’s wort) on the one hand, and anticancer drugs on the other [228], or the interaction between rifampicin and retrorsine (a pyrolizzidine alkaloid occurring occasionally as a contaminant in herbal teas) [229]. A PBPK model was used to show that cichoriin is capable of reaching high concentration levels in the lung (when administered intravenously), and thus, that it is a potential candidate for development against COVID-19 [230] and can predict the plasma concentration variation for schaftoside, subsequent to the ingestion of a capsule containing total flavonoids extracted from *Desmodium styracifolium* [231].

## 3. Limitations of in Silico Methods

Although computational methods are promising and provide multiple advantages, from low cost to high speed, their usefulness is still impacted by significant limitations that users must be cognizant of. Certain limitations apply broadly to many or all computational methods, while others are unique to specific approaches. For instance, all methods exhibit a dependence on data quality and quantity, all need experimental validation/confirmation, all tend to use static or oversimplified representations of the real chemical or biological processes, failing to capture fully the dynamic biological environments. In addition, each method comes with its own specific limitations—for instance, the risk of overfitting, a phenomenon describing a very good performance of a model on the training data but a poor performance on new data, not previously seen by the model, tends to be specific for QSAR models. We first review the main general limitations and then discuss specific limitations for each of the methods discussed in this paper (a synthetic presentation of the latter is presented in Table 3).

### 3.1. General Limitations

(a) Dependence on data quality and quantity. Most in silico models (including those used for PK purposes) are heavily dependent on the quality of the initial data, as well as their volume. This is due to the nature of those models, but research confirmation investigating specifically the influence of data on model results are available in the literature [232]. For instance, QM/MM methods, which are otherwise physics-based, require accurate initial structures of the protein–ligand complex (generated experimentally with X-ray crystallography, cryo-EM, or NMR) [233], mechanistic studies to understand a reaction steps, transition states, reaction intermediates, etc. [234,235] Often QM/MM methods are applied to data generated in MD simulations, and in such a case, if the MD simulation has been impacted by a structure error or missing atom, it will get propagated further to the QM/MM predictions [236]. Ligand–protein docking results are also impacted not only by computation algorithms but, to a good extent, by initial experimental data, as follows: the protein structure (as confirmed by X-ray crystallography, cryo-EM, or NMR) and the known binding site (or predicted on the basis of other experimental data for other proteins) are dependent on the quality of the experimental data. Even the scoring functions used in molecular docking are often empirical, being built by training on experimental data sets or knowledge-based functions, derived from 3D-structures of protein data sets, although “pure”, physics-based scoring functions are also available [237,238]. QSAR models are by their nature directly dependent on the quality and quantity of the training and testing data sets [239], and the situation is very similar for pharmacophore [98] and PBPK models [240].
(b) Actual performance in laboratory or living systems of a drug active ingredient may often be influenced by additional factors that in silico models have not taken into account [238]. For instance, an oral absorption prediction model might predict high absorption based on passive diffusion and membrane permeability, but if the model ignores an active efflux mechanism (involving the Pgp, for instance), it might wrongly overpredict oral bioavailability. Likewise, a model might undervalue the importance of uptake transporters such as OATPs, which are often involved in hepatic drug uptake [241], or overlook differences in CYP enzyme activity caused by genetic variations between individuals [242]—both of which can result in incorrect estimates of how drugs are absorbed or eliminated. About 20% of the compounds in a data set of 117 substances showed over 5-fold lab variability in the measurement of the fraction unbound to human plasma (with a maximum 185-fold); if a prediction is based on a low value of the range, for instance, it could result in important errors in prediction, not because the input data was wrong in itself, but because the input data were incomplete and ignored large values that could equally be found in the real world [243].
(c) All in silico tools need experimental validation to be reliable and applicable in real-world contexts. “Dry data” generated by computers should be confirmed by “wet data” generated in wet lab experiments [244]. It is not always easy to have the validation by the same scientists (often the computational researchers do not have the tools or expertise required to perform the wet lab investigations), but non-validated data generated with computational means should always be regarded as only hypothetical until confirmed (or not) experimentally. In the case of QM/MM models, it has been argued that seemingly accurate predictions against experimental data can sometimes be the result of merely reciprocal cancelation of errors, for instance, when limitations in the QM approach and a small QM region offset each other [245] (for instance, if the QM component overestimates binding energy by 5 kcal/mol due to an imperfect approximation, while the MM component underestimates it by 5 kcal/mol due to an oversimplification, the errors cancel out—producing a result coherent with experimental data; however, the model could fail in other systems where errors do not happily balance as in this hypothetical case).

### 3.2. Specific Limitations

QM or MM models have multiple limitations that are specific to these in silico approaches. In the case of QM, probably by far the most important limitation is related to the computational costs, as they need computer resources and long periods of time [20]. In the last two decades, great progress has been made through improvements in both computational resources (including GPU acceleration and cloud computing) and new methodologies that have made QM estimations to larger and larger systems and a wide variety of applications increasingly accessible, including in the field of biology, drug discovery, and more specifically, pharmacokinetics. Exact solutions to the core equations of quantum mechanics are limited to a handful of special and rather simple cases. For other systems, including biological ones, one must rely on approximations, numerical methods, or both. Therefore, there are cases where an empirical method, optimized for a particular property, could outperform calculation based on a pure QM approach [246]. Another limitation derived from this refers to the fact that whereas QM is focused on events at atomic or molecular level, PK phenomena often take place in much more complex environments (large proteins, membranes, entire cells), which are more difficult to model at atomic level due to the high computational costs. For instance, running full quantum mechanics calculations on cellular membranes is still beyond our current capabilities [247]. Quantum mechanics is highly effective for modeling molecules in fixed states, but pharmacokinetic processes require understanding dynamic fluctuations that occur as drugs move through biological systems and undergo various chemical transformations [248], and this creates another limitation for QM applications for PK predictions. In the same line, whereas PK process takes minutes and hours, QM simulations are limited to picoseconds or at best, nanoseconds. QM is also limited in the scope of PK applications that can be estimated with it. For instance, one cannot simulate/predict directly the oral absorption of a drug with QM, but the latter can help with the computation of certain descriptors (e.g., logP) that could explain more or less the permeability of a substance [249]. Similarly, QM cannot predict whether a drug will bind to human serum albumin, but QM/MM could be used for such a prediction [250]; neither QM, nor QM/MM approaches, though, could predict the tissue distribution of a drug (of natural or synthetic origin) or of its metabolites.

QM/MM, although more efficient than pure QM methods, still remain computationally costly, and although progress has been constant with respect of both computational resources and algorithms, the complexity of the molecular systems in biology (and, in particular, in PK) make extensive sampling necessary, which remains challenging even with lower-cost methods [251]. The choice of the QM/MM boundary, if not well defined or smoothly treated, risks creating artifacts because of the spatial discrepancies in how interactions are represented (especially solvent structures, as hydrogen bonds between water molecules, for instance, can be over- or underestimated). Such artifacts have stronger consequences in dynamic than in static simulations, but even the latter are not fully immune from their effect [252]. Reaction energetics (barriers and energies) tends to show little dependence on the QM region size, whereas the charge transfer between the solute and its surroundings converges more slowly, larger QM regions being necessary [253] and thus, more computational power is required. If the charge transfer is not correctly estimated in ligand–protein interactions, this can impact PK predictions, such as membrane permeability. The short timescales, the poor parametrization [254], and the limited or poor capturing of entropy due to the limited sampling [255,256], all can also impact the accuracy of the QM/MM estimations. By reducing simulation costs and accelerating dynamics, coarse graining (discussed below, in the paragraph on MD simulations) is a method known to enhance sampling efficiency. Therefore, integrating a QM system within a CG environment promises to accelerate sampling while retaining detailed information about the QM region [257].

Ligand docking is also subjected to multiple limitations that can impact its performance, outlined as follows: limited treatment of flexibility (most often the protein is modeled as rigid or semi-rigid) often ignoring induced fit, reliance on simplified scoring functions (which may poorly capture complex interactions, resulting in prediction errors), inadequate treatment of solvent effects, [258] or poor treatments of entropy contributions [259]. Although ligand docking is efficient computationally with smaller- or medium-sized systems, in the case of large conformational spaces and for a large number of compounds, it can still be demanding in computational terms. Flexible and induced fit docking is much more computationally expensive than rigid docking [258]. Ligand docking is often performed, and its results are reported in a sub-standard manner. Even in high-impact publication docking, results of a questionable quality can be encountered. This may be the result of the target protein’s binding site being poorly identified, using an unsuitable ligand database for screening purposes, inappropriate decisions in choosing the docking pose, a high predicted binding affinity (docking score) that is not confirmed by MD simulations, confusion about whether the compound is an inhibitor or an agonist, or docking results that are contradicted by experimental bioassays [260]. The mere docking score is not in itself sufficient to assert that a ligand is an agonist or antagonist, as it only indicates the binding affinity of the ligand; the only way to clarify this aspect is usually to perform wet lab bioassays [260].

The primary limitation of pharmacophore models is the absence of a general, effective scoring method, unlike docking, where, as discussed above, there are multiple scoring functions available, with different approaches. Most often the degree to which a ligand fits a pharmacophore query is estimated by RMSD, but this metric does not take into account similarities to known inhibitors nor can it predict the overall compatibility. Consequently, compounds that hit perfectly a pharmacophore query might differ significantly from other inhibitors and could, however, have functional groups incompatible with the receptor’s binding site, resulting in their inactivity [261]. Following the same idea, if the model is oversimplified (the number of key features selected is too small), it will result in many false positives [262]. A second important limitation consists of the fact that the pharmacophore model depends on databases that store a set of pre-generated low-energy conformations (3D shapes) for each compound. Although multiple conformations may have similar energies, only a small number are stored in the database. Therefore, a potential hit may be missed during the search because the specific conformation is missing. To avoid this, many pharmacophore software applications are programmed to rotate flexible polar bonds during the search process, so as to avoid missing such active compounds with rotatable polar bonds [261]. Similarly, the same molecule can exist in multiple tautomeric forms (e.g., keto-enol) or protonation states (e.g., charged vs. uncharged). A pharmacophore model built with the wrong typing (e.g., the charged instead of the uncharged form) will result in wrong matches in the screening process [263]. Good quality models are built starting from ligand–protein crystal structures; if such X-ray structures are lacking, the key interaction sites (known as anchoring points) will have to be guessed, but if incorrectly chosen, the model will fail in its results [263]. For most data sets, usually there are multiple ways in which a pharmacophore can be built (there is no canonical algorithm of building them). Hence, different pharmacophore models can be built for the same data set, and experiments have suggested that such models can select very different candidates when applied in the virtual screening process [261]. Traditionally, pharmacophore models have been static in nature, whereas the ligand–receptor interaction is dynamic; more recently attempts have been made to build models by considering the dynamic nature of those interactions [264].

As discussed in the general section, QSAR models, like other in silico approaches, depend on the high quality and sufficient input of data. A primary limitation specific to QSAR is overfitting, where models perform well on training data but poorly on new compounds due to learning noise rather than true structure–activity relationships. This reduces model generalizability, particularly for compounds not encountered during training. Even high statistical correlations do not guarantee causality, and mechanistic interpretations are recommended to enhance model credibility [265]. More recently it has been argued that “nowadays all models can be considered interpretable” [266]. A high Q^2^ value is a required condition, but not sufficient on its own to guarantee predictability [267]. A key limitation of QSAR models is that their prediction of a biological response is valid only if the compound being predicted is within the so-called “applicability domain” (AD) of those models, i.e., a theoretical chemical space in which the models produce reliable predictions; AD is defined by the descriptors used by the models, the modeled response, and the features of the chemical compounds forming the training data set [265,268]. When closely related chemical compounds exhibit vastly different biological activities despite nearly identical structures, they create what it is called ‘activity cliffs’. They pose significant challenges for QSAR modeling, often creating prediction errors that exceed those caused by inherent limitations of in silico models [111].

For MD simulations, a first important limitation is the imperfect nature of the currently used force fields (mathematical models describing atomic interactions), which need further refinement and improvement to better represent the real particle behavior; the polarizable force-fields remain a particular challenge [213,269,270]. A second key limitation of this approach relates to the high computational demands, which restrict simulations to small lengths (nanometers) and time periods of microsecond timescales at best, most often nanoseconds or even femtoseconds for very large systems (simulations up to a few milliseconds are now possible, but they are hardly accessible to most researchers). For instance, even a relatively small system (25,000-atom) requires several months of computation on non-trivial hardware (24 processors) for a single microsecond of simulation time. The short times typically used may provide insufficient sampling of important conformational states that molecules can adopt within biological processes relevant for PK purposes, hampering the study of molecular phenomena of longer durations or of larger structures, such as protein folding or unfolding and protein interactions with membranes [269,270,271,272]. To solve this challenge many enhanced sampling methods have been developed in the last three decades, which can accelerate the exploration of rare conformations and increase the likelihood of detecting low probability, high-energy events, potentially relevant for biological reactions/processes [271]. An alternative to conventional all-atom MD is the coarse-grained MD, which simplifies a system by combining groups of atoms—typically from 4 to 6—from the protein, ligand, or the solvent into single particles called “beads.” For example, the Martini force field represents four water molecules as one bead. This significant reduction in the number of particles and complexity smooths the interaction energy surface and makes it feasible to simulate larger systems over extended timescales at the cost of some loss in atomic detail [270,272]. MD simulations are not well-suited for systems where quantum effects play a prominent role, e.g., in cases involving transition metal atoms in binding processes, in such cases, QM/MM hybrid approaches are preferable [269]. MD simulations of membrane proteins often exclude the membrane due to computational costs, but biological membranes influence significantly the drug action by modulating substrate selection and receptor function across various membrane protein targets (polytopic, bitopic, and peripheral), hence this is an important limitation [270].

Predictive PBPK models tend to overestimate time-dependent inhibition (TDI) of CYP3A4 substrates and to underestimate CYP3A4 induction. When an experimental drug exhibits both TDI and induction in vitro, a PBPK model may incorrectly predict the result, indicating net inhibition when wet lab DDI experiments show net induction [221,240]. Conducting in vitro in vivo extrapolation (IVIVE) using PBPK models generally demands a significantly greater volume of experimental and computational data than static models. Therefore, the reliability of model predictions can be compromised due to uncertainties in individual parameters. For instance, the distribution and absorption parameters cannot be experimentally validated or verified if a modeled compound has never been administered intravenously in humans, and using an extrapolated or predicted value (from an in silico model) will infuse uncertainty into the model parameters and outcomes [217]. There is very limited experience with PBPK modeling and in vitro in vivo extrapolation (IVIVE) for non-CYP enzymes such as UDP-glucuronosyltransferases (UGTs) or sulfotransferases (SULTs), and others, but examples are starting to emerge. Assessing drug–drug interactions mediated by such enzymes presents several difficulties, including limited knowledge of genetic variations (polymorphisms, e.g., for UGTs,), of transporter involvement in the PK of certain drugs and of extrahepatic drug elimination [217,221,273]. The commercially available PBPK software, although improving, does not include all the variables that should be included in certain models and need improvement or customization; programming skills are therefore often needed, depending on the product and pharmacological context modeled (e.g., excipients, dosage form, route of administration, etc.) [217,274]. Along the same line, more physiological parameters could/should be included in the building of many models, to better reflect the biological mechanisms involved in the PK of many compounds [274]. PBPK modeling for therapeutic proteins remains limited in utility due to inconsistent model structures and overparameterization risks from sparse target expression data [217], but it is expected that with more experience and data, this will improve in the future. The best way in which PBPK models should be validated is still undecided [217]. For a number of areas there is very limited experience with PBPK models, as follows: populations with organ impairment; applying allometric scaling to infants and toddlers (<2 years); and geriatric, obese, and pregnant patients [240]. Another significant limitation of PBPK is its limited capacity to determine tissue-specific distribution in humans. This issue has been to a good extent solved through studies that connect tissues’ affinity for drugs to their composition and physicochemical properties [275]. PET imaging also promises to improve building PBPK models and their validation in terms of tissue distribution [276].

## 4. Is the Performance of in Silico Models Confirmed by Experimental Data?

Validating computational models for natural compounds is not a one-size-fits-all process—it depends on the computational method used, although many aspects are common or similar. Quantum mechanics favors mechanistic accuracy, comparing predictions like bond energies and stereoselectivity directly with lab results. Molecular dynamics simulations require experimental confirmation of predicted molecular behavior depending on the nature and purpose of the simulation. PBPK models often use fold-error analysis with a two-fold acceptance threshold for key pharmacokinetic parameters (Cmax, AUC), while QSAR models must meet minimum statistical thresholds of q^2^ > 0.5 and r^2^ > 0.6, as well as “predicted versus experimental values” plots, though performance usually exceeds these minimal criteria in published papers. Docking validation looks for agreement between computational scores and experimental binding data (IC50/Ki values), whereas pharmacophore models are validated through prospective experimental testing, depending on the purpose and target of the model.

Prospective validation is the gold standard, but due to practical constraints, most studies rather rely on existing data, using retrospective validations. All approaches must take into account the structural diversity and target specificity and proper validation of new models should always be carefully performed. Experimental validation of in silico predictions for natural compounds in the field of PK using diverse tools is limited, but there are documented instances in the literature where such predictions have been confirmed for certain natural compounds, which we review here (and are summarized in Table 4).

### 4.1. QM and QM/MM Methods

Experimental confirmation for QM predictions are rarer for natural compounds (and also for synthetic derivatives), because QM techniques are often used to rather understand the mechanistic aspects of processes already established through wet lab experiments. For instance, QM computations have been used to obtain insights into how piperine—present in different types of pepper—enhances the bioavailability of different drugs, including a boost in the absorption of curcumin by approximately 20-fold [294].

Similarly, starting with experimental data for CYP3A4 inhibition by several flavonoids (chrysin, pinocembrin, acacetin, and apigenin), Mitrasinovic (2021) used a QM/MM model (SCC-DFTB-D/AMBER) to estimate the binding free energies of those compounds to the CYP3A4 and found a strong correlation with the experimental values. Chrysin was the most potent inhibitor both experimentally and computationally and was followed by pinocembrin. Acacetin and apigenin exhibited weaker inhibition both experimentally and in silico, and this was related to less favorable estimated binding free energies [295].

In another study, the computed overall stereoselectivity for the trans-5′-hydroxylation of (S)-(−)-nicotine by CYP2A6 (using a QM/MM free energy method) was approximately 97%, and this was in good agreement with the stereoselectivity of 89–94% estimated earlier in wet lab experiments [277].

LogP values estimated with the AM1 QM method have been used to classify a number of 120 pharmaceutical compounds according to their ability to cross the BBB. Among the compounds predicted to cross BBB was caffein, a natural compound well known for its ability to readily reach the brain and exert its psychopharmacological effects [278]. Thus, the LogP value for caffeine can be regarded as confirmed by clinical data. For other, non-natural compounds analyzed in the source cited, experimental data were also in agreement with the computed values.

DFT calculations were used in a study to identify the major catalytic site in CYP450 by estimating C-H bond dissociation energy in 50 relatively simply chemical compounds, some of which are found in nature (e.g., acetic acid, acetic acid methyl ester, allyl alcohol, trimethylamine, etc.) and comparing it to experimental data. All compounds and their neutral radicals were optimized using B3LYP/6-31+G(d,p), as implemented in Gaussian 09. They found that the average C-H bond energy dissociation at the main metabolic site was statistically lower by 6.9–12.8 kcal/mol as compared to other C-H moieties [279].

Ahmad et al. (2023) used DFT to estimate the global reactivity parameters (GRP) of 4-hydroxyisoleucine (an aminoacid isolated from the seeds of *Trigonella foenum-graecum* L.), and for all isomers they predicted a reduced chemical reactivity and relatively high stability [296]. They have not experimentally evaluated this prediction, but an independent study carried out three years earlier has found that for an LC/MS PK method, the compound is stable in plasma under different conditions typically evaluated for this type of studies [297].

### 4.2. Molecular Docking

Molecular docking is also often employed to better understand molecular interactions that have already been observed in wet lab experiments. For instance, molecular docking has been used to enhance the understanding of experimental in vivo findings related to the inhibitory effects of garcinol on various CYP P450 enzymes. In vitro data revealed that garcinol exhibits strong inhibition against several CYP enzymes, with IC50 values indicating potent inhibition for CYP1A2, CYP2C9, CYP2B6, CYP2D6, and CYP3A4. To deepen the understanding of these findings, molecular docking was conducted to investigate the interactions between garcinol and the active sites of CYP2B6 and CYP3A4 proteins. The results indicated that garcinol binds in the active sites within these enzymes, leading to interactions with critical residues that result in the enzyme inhibition [298].

At 10 µg/mL, curcuminoids suppressed both the 17α-hydroxylase and 17,20-lyase functions of CYP17A1. CYP21A2 activity was only marginally affected, whereas CYP19A1 activity decreased by up to 20% in a dose-dependent manner across concentrations of 1–100 µg/mL. Molecular docking studies confirmed that curcumin can interact with the active sites of CYP17A1, CYP19A1, and CYP21A2 [299]. This approach of using docking to better understand experimental findings was used to gain insight into the interaction between berberine and Pgp [300], a series of flavonoids and CYP2C9 [301], silybin and CYP2B6 [302], resveratrol or pterostilbene and human serum albumin [303], berberine and MATE1 or Pgp transporters [304], and others [44].

Proper experimental validation of molecular docking prediction has been very rare in the literature up to date. Handa et al. (2014) performed a retrospective validation of their docking procedures used to predict the interaction of several drugs with the following two variants of CYP2D6: CYP2D6.1 (wild type) and CYP2D6.17 (a natural variant expressed primarily in African populations). They rescored the top 30% Glide docking scores with the MM-GB/SA method and built a regression equation describing the pKi values as a function of the MM-GB/SA scores. For both CYP2D6.1 and CYP2D6.17, there was excellent agreement between the predicted and measured values, with correlation coefficients estimated at 0.81 and 0.92, respectively [280]. This can be considered experimental validation, but it is retrospective in nature, not prospective.

A similar retrospective validation was used by Daddam et al. (2014), who compared the docking scores obtained for the binding of 10 flavonoids to Pgp and the IC50 values reported in the previously published literature. The authors claimed that “the inhibitory activity (IC50) and docking results of other Flavonoids were corelated…. It can be concluded that docking results are in correlation with inhibitory activity of flavonoids” [281]. However, the correlation coefficient between the two variables (docking scores and IC50 values), if computed, is low (r = −0.27 with the Pearson method and r = 0.079 with the Spearman method) and statistically non-significant (*p* = 0.44 and 0.84, respectively).

As mentioned in the section on molecular docking, Marques et al. (2021) created a dynamic ensemble of Pgp structures to investigate the binding modes of lignans known to inhibit Pgp. The docking results were statistically analyzed to identify a representative Pgp structure that could effectively distinguish between active and inactive compounds. Statistical analysis of the docking results led to the selection of a system for virtual screening of potential P-gp inhibitors. The method was tested on a library of 87 natural flavonoids, with 10 being experimentally tested. Their results only partially matched the theoretical predictions, but at least two flavonoids exhibited properties of P-gp inhibitors, enhancing doxorubicin’s accumulation and antiproliferative activity in overexpressing Pgp cells [51].

Isca et al. (2021) used molecular docking, molecular dynamics, and other computational methods to assess the effects of a number of abietane diterpenes from *Plectranthus* spp. (Lamiaceae), as well as several hemisynthesis compounds on Pgp, and to understand the molecular mechanisms by which they inhibit the transporter protein. Docking simulations indicated that aromatic groups enhance the P-gp binding affinity in royleanone derivatives, and one benzoylated derivative appeared to act as a noncompetitive modulator of the transporter by acting at the M-site. The authors experimentally validated their findings by evaluating rhodamine 123 accumulation in multi-drug resistance NCI-H460 lung carcinoma cells, but this was performed only on two of the hemisynthesis compounds [282].

### 4.3. Pharmacophore Models

Pharmacophore models demonstrate varied experimental performance in their prediction of biological activities, including PK properties. For instance, in the study of URAT1 inhibitors, a combination of four pharmacophoric models with other computational techniques successfully narrowed down 11,000 natural compounds to 25 flavonoids. While in vitro evaluation identified fisetin, baicalein, and acacetin as inhibitors, their IC50 values (12.77–57.30 µM) indicated relatively low potency [105].

As discussed in the section on pharmacophore models, Hochleitner and colleagues (2017) used a previously developed pharmacophore model to identify new CYP2D6 inhibitors and identified 75 hits among a database of natural compounds. Subsequent in vitro testing of 23 candidates (including the luciferase inhibitor resveratrol as a control) exhibited strong CYP2D6 inhibition in 10 compounds (42%), moderate inhibition in 8 (33%), and no activity in 6 (25%), indicating good reliability for the pharmacophore model and the proposed workflow [110].

Kaserer et al. (2015) used a variety of in silico models to predict DDIs produced by inhibition of CYP1A2, 2C9, and 3A4 enzymes. They tested the models on 29 compounds, both synthetic and natural. Among the computational tools evaluated in this study, they included pharmacophore models developed with several tools, as follows: LigandScout, PharmMapper, and Discovery Studio. The predictions were compared with in vitro results obtained with fluorescence-based P450 microarrays. The LigandScout models had accuracy rates of 65.5% for CYP1A2, 55.2% for CYP2C9, and 53.6% for CYP3A4. For PharmMapper the accuracy rates were 55.2% for CYP1A2, 48.3% for CYP2C9, and 32.1% for CYP3A4; for two of the three enzymes, the PharmMapper models made no predictions among the 29 compounds (all predictions were negative; therefore, the accuracy rates coincided with the true negative rates), but the authors were not able to estimate whether pharmacophore models for the latter two enzymes were included or not. For the Discovery Studio pharmacophore models, the estimated accuracy rates in this study were 51.7% for CYP1A2, 65.5% for CYP2C9, and 64.3% for CYP3A4. In this study the accuracy of pharmacophore models for CYP450 prediction varied by isoform as follows: CYP2C9 showed the highest overall accuracy, followed by CYP3A4, while CYP1A2 predictions were the least consistent, with moderate accuracy in LigandScout (65.5%), low in Discovery Studio (51.7%), and no predictive capability in PharmMapper [283].

Wang et al. (2017) used pharmacophore modeling and molecular docking to identify potential mechanism-based CYP3A4 inhibitors among 105 compounds from Tripterygium wilfordii (Celastraceae). Their pharmacophore results suggested that only five compounds would be such CYP3A4 inhibitors, and molecular docking results narrowed their number to the following three: wilfortrine, wilforgine, and euonymine. In vitro enzyme inhibition assays revealed moderate inhibitory effects of these three alkaloids on CYP3A4 activity, whereas wilfordine and wilforine (indicated by the pharmacophore model but not by docking) showed no important inhibition. These experimental outcomes were consistent with the predictions of the computational models, validating the predictive accuracy of their combined approach [284].

Yang et al. (2012) used molecular docking and two pharmacophore models to screen 56 compounds of herbal origin to identify potential CYP1A2 inhibitors. Molecular docking identified 19 compounds, and the pharmacophore models narrowed them to 12. Using in vitro experiments, the authors found that 7 of the 12 compounds were moderate to potent CYP1A2 inhibitors. Thus, a combined approach based on both docking and pharmacophore screening had an overall prediction accuracy of 58% [285].

### 4.4. QSAR Models

QSAR models are only acceptable if appropriately validated against experimental data. As recommended by one expert in the field, in scientific papers, QSAR models should be described “not only with their statistical performances, but also with the plot of predicted versus experimental values” [305]. Therefore, in the case of QSAR, even with imperfect validation there is some evaluation of performance against experimental results. This validation is retrospective in nature and obviously prospective validation would be preferable, but often the builders of the model(s) are not in the position to perform such a prospective validation. Although for regression models minimal acceptance criteria are q^2^ > 0.5 and r^2^ > 0.6 [239], in practice, the performance is over this minimal threshold.

Thus for COMFA and COMSIA models developed in one study for a variety of natural phenolics, good performance was reported in retrospective validation (r^2^pred 0.78 and 0.70), but critics argue that aligning structurally diverse molecules impacts the reliability of the 3D models, “considering that molecular alignment is a crucial step in COMFA and COMSIA” [163].

In the case of intestinal absorption prediction, the performance of QSAR models evaluated in a large benchmark study was only slightly inferior to that of in vitro methods (83% of QSAR predictions and 87% of in vitro method predictions fell within 2-fold of observed values) [286]. Similarly, an earlier study indicated that a pair of computer models achieved reliability comparable to the Caco-2 and 2/4/A1 cell lines, with one model predicting the absorption of a collection of 65 medicines nearly as well as in vivo absorption experiments conducted in rats [287].

### 4.5. Molecular Dynamics

Studies of molecular dynamics simulations in the PK field, where predictions have been experimentally validated, have also been scarce, but one excellent example is that of Wadhwa et al. (2021), who evaluated the ability of withaferin-A and withanone—two structurally analogous withanolides with markedly different cytotoxic profiles—to permeate a model bilayer membrane. The simulations indicated that withaferin-A exhibits substantial transmembrane transport, unlike withanone, whose transfer is predicted to be significantly impeded. Analysis of free energy profiles showed that the membrane’s polar head groups present a major energy barrier for withanone, while both molecules experience comparable environments within the membrane interior. The accuracy of the MD simulation predictions was evaluated experimentally by using specific antibodies tailored to react with the two compounds. Timelapse imaging of both untreated and treated cells showed that withaferin-A penetrated the cells to a higher degree than withanone, a finding in agreement with the computational predictions [288].

Using molecular docking and molecular dynamic simulations, Patil et al. (2022) found that curcumin and quercetin bind to the CYP3A4 protein and displace CDK inhibitors (palbociclib and ribociclib), and they experimentally validated their findings through in vitro assays. They found consistent IC50 values for the following two natural compounds: 16.10 μM for curcumin and 0.05 μM for quercetin. They also experimentally found that the two CDK inhibitors exhibited a 4.4–9.9-fold increase in half-life when incubated with curcumin or quercetin, as compared with their testing in the absence of the two natural compound, because of the reduced clearance rate of the two compounds. Thus, experimental findings were in good agreement with the in silico predictions [289].

### 4.6. PBPK Models

For PBPK models, there is more experience with synthetic drug ingredients than with natura compounds, but limited experimental validation is also available for compounds of natural origin. For example, in one paper reporting PBPK data for several natural compounds, the authors, to evaluate the accuracy of the dose estimation of oxymatrine (a major Sophora alkaloid) by the PBPK model, compared the predicted oxymatrine dose against its clinically recommended dose. The predicted dose was 367 mg 3× daily, whereas in the clinical practice it is administered doses of 200–300 mg 3× daily, a difference considered as relatively small and found acceptable by the authors [290].

To verify a PBPK model’s ability to predict DDIs of hyperforin with sedative-hypnotics in human patients, Shin et al. (2024) compared the predicted AUC ratios for zolpidem, alprazolam, and midazolam against those observed in clinical studies. The predictions were in good agreement with the clinical data, as follows: for zolpidem, the observed AUC ratio was 0.7 compared to a predicted 0.8; for alprazolam, 0.97 vs. 0.96; and for midazolam, 0.2 versus 0.22. All predictions were deemed to be within an acceptable margin of error [291].

A PBPK model to predict the PK of hydrastine and berberine after a single dose of goldenseal extract was verified by comparing the predictions with clinically observed data. The predicted plasma concentrations were generally in agreement with clinical observations, with most clinical values falling within the 5th to 95th percentiles of the predictions, except for berberine Cmax, which was slightly overestimated. The predicted AUC values for hydrastine and berberine were also close to the clinically reported values (the predictions were 15% higher and 9% lower, respectively). The model also predicted a clinically significant interaction with midazolam but not indinavir, consistent with clinical data. The predicted Cmax values for midazolam and indinavir were slightly higher than the clinically observed values but still within an acceptable range. The model also predicted that hydrastine’s inhibition of ABCB1 would lead to a slight elevation in digoxin’s Cmax (a simulated ratio of 1.23 versus the clinically observed value of 1.14). The predictions regarding the interactions between a high dose of berberine (300 mg 3× daily) and CYP3A substrates, such as midazolam and cyclosporine A, were also consistent with the clinical pharmacokinetic data [292].

A PBPK model for piperine was validated by comparing the predictions with the actual clinical data. The model used oral piperine doses of 20 mg (single administration) and 20 mg daily for one week (repeated doses) in healthy Chinese participants. The model exhibited strong predictive performance, with prediction errors of 1.3-fold and 1.4-fold for Cmax and AUClast in the single-dose simulation and 1.0-fold and 1.1-fold for the repeated-dose simulation. Since all error values were below the two-fold acceptance criterion, the PBPK model was considered valid [293].

A key challenge common to all methods discussed above is their reliance on retrospective validation. Although published models tend to demonstrate strong agreement with available experimental data, genuine prospective validation—where computational results directly inform new experiments—is still rather uncommon. This limitation poses a major barrier to the broader integration of in silico approaches in natural product drug discovery, and, in particular, in understanding the PK properties of natural compounds.

As illustrated by the few cases relevant to our theme, validation studies seem most effective when they integrate several computational approaches. Specifically, combining pharmacophore modeling with molecular docking consistently yielded better predictions in the published literature than using either method alone. This suggests that incorporating diverse approaches improves the reliability of predictions and should inform how future models are developed and applied in drug development.

The available data, while limited in volume, indicate that model performance varies significantly based on the specific biological target and natural compound structural class. Since the published cases in the field of PK for natural compounds are rather scarce, it is imperative that more research is carried out to obtain a better understanding of the relationship between the performance of different in silico models and biological targets and natural compound structures.

## 5. Comprehensive ADME Tools

Whereas many of the approaches discussed above involve the building and validation of a single model or a family of models that are then used “inhouse” for the virtual screening of natural or synthetic compounds, there are a number of software tools that have been developed specifically for in silico ADME predictions. They are either available as server services or as standalone products, for free or for a fee. Although a larger number of commercially or freely available software is available for ADME predictions, our search of PubMed using appropriate keywords (“natural” OR “herbal” plus the name of each software product, and when not very specific, also the name of the manufacturing company, for all years up to March 2025) has shown that for natural products only a part of the available software has been used, and only a few have been applied in multiple papers in the field of natural products, outlined as follows: SwissADME webserver [306] (>250 papers), pkCSM [307] (>60 papers), ProTox-II/Protox 3.0 [308,309] (>45 papers), QikProp [310] (part of the Schrödinger Suite > 40 papers), ADMETSAR [311] (>39 papers), ADMETlab 2.0/3.0 [312,313] (>30 papers), ADMET Predictor [314] (>12 papers), FAF-Drugs4 [315] (6 papers), ACD/Percepta [316] (4 papers), and the ADMET module of Biovia Discovery Studio (2 paper [43]). Admetboost [317] and Interpretable-ADMET [318] have been relatively recently published and made available for free, but no paper has apparently used them as of yet in the assessment of natural products.

Most of the mentioned tools are web-based and freely available (SwissADME, pkCSM, ProTox-II/Protox 3.0, ADMETSAR, ADMETlab 2.0/3.0, Admetboost, Interpretable-ADMET, FAF-Drugs4), whereas a few are available as standalone or modules of standalone applications (QikProp and P450 SOM modules of Schrodinger, ADMET Predictor, ACD/Percepta, ADMET module of Biovia Discovery Studio). Other tools are also available, although they seem to have not been used in the assessment of natural products yet, at least on a significant scale; one such important tool is NERDD, which provide a set of several web tools useful in the ADME prediction of both natural and synthetic compounds [319]. A review of 18 freely accessible web-based tools for ADMET prediction, covering their pros and cons, model-based calculations, and degree of accuracy, has been recently reported in the literature [320].

We have limited the discussion to general-purpose ADME tools, but specialized tools for specific ADMET are also available; for instance, a review of software applications used to specifically predict CYP450-mediated drug metabolism is available elsewhere [321].

A synthetic comparison of the four main free ADME web-server applications is shown in Table 5.

## 6. Conclusions and Future Perspectives

The application of in silico ADME-PK methods in pharmaceutical research became widespread following Lipinski’s rule of 5, though it has historically been viewed as an auxiliary component of disciplines like drug metabolism, pharmacokinetics, and computational chemistry. Most practitioners in this field come from backgrounds in computer science, chemistry, or drug metabolism and PK rather than having specialized education in in silico ADME-PK techniques. While this lack of formal training might seem limiting, it has actually fostered a collaborative environment where experts from diverse fields—including in vitro research, statistics, analytical chemistry, pharmacokinetics, structural biology, medicinal chemistry, and machine learning—can work together to drive innovation [322].

Initially met with some hesitation, the application of in silico ADME models and tools to natural compounds, particularly those of herbal origin, has witnessed a significant surge in recent years. While this field is relatively young, having emerged within the last three decades, its growth has been remarkable. The number of research publications utilizing in silico approaches for assessing natural compounds has surpassed 3000 in our estimation, with a substantial majority appearing within the last five years. This rapid growth is evident in the following publication trends:

- Pre-2020: Fewer than 100 publications containing the phrase “in silico” in the title or abstract.
- 2021: Approximately 200 such publications.
- 2023–2024: Over 270 such publications annually.

Similarly, if only seven papers indexed by PubMed seems to have included “natural” and “docking” in their title or abstract before 2010, in 2024 alone the number of this kind of papers has exceeded 300. This exponential growth underscores the increasing recognition and adoption of in silico methods in natural product research.

As seen in this review, there are multiple ways of approaching ADME predictions using computational methods, each with their own challenges, costs, and limitations. Quantum mechanics methods have been less used, but it is likely that their applications will also increase more, as is already evidenced by the trend in numbers in the last ten years. The usefulness of molecular docking in speeding drug development remains a topic of debate, despite the significant refinement and improvement of this method over the years. It is widely known that the predictive accuracy of docking often falls short in complex biological systems and it is expected to improve the currently available docking tools to make them more reliable. Even so, molecular docking continues and will probably continue to play a valuable role, particularly in the field of natural products, where resources for wet lab experiments are scarcer. One would expect that pharmacophore modeling will also improve, moving beyond the conventional approach based on key interaction points to more sophisticated approaches, for instance, integrating quantum mechanics calculations or and machine learning tools. This will allow a better understanding of pharmacophoric features particular to natural compounds and better predictions, not only of pharmacodynamic interactions, but also of their ADME profiles.

The increasing computational power and refinement of force fields is likely to allow more accurate predictions of protein–ligand interactions, membrane permeability, and metabolic transformations for natural compounds in the future. The extension of these techniques and improvement with the help of machine learning approaches will probably contribute even more to a better understanding of protein interactions with natural compounds. They might even be successful where traditional QSAR methods may fall short due to their structural complexity, novelty and diversity. However, QSAR methods, particularly in implementations based on deep learning approaches seem to be the de facto workhorse for ADME predictions, particularly in the case of ADME(T) prediction servers. An impressive number of such servers have become available for free in the last decade, and they seem to be particularly valued by natural compound researchers, who tend to use them increasingly more frequently in their research.

Finally, the continued advancement of -omics technologies, coupled with the generation of additional wet lab experimental data, is expected to refine and improve predictions of natural compound ADME properties using PBPK modeling. All of these methods will most likely continue to evolve and improve, and they will allow for a better understanding of ADME profiles and druggability, not only for natural compounds but also for various chemical modifications of such compounds, specifically intended to make them more appropriate for drug development purposes.

The field of pharmacokinetics/ADME provides a powerful example of a virtuous cycle where the continuous generation of experimental data drives the improvement, refinement, and expansion of computational prediction tools, which in turn provides insight and favors experimental design and additional lab data generation. Public databases such as the FDA’s Liver Toxicity Knowledge Base (LTKB) and ChEMBL have and will continue to stimulate new method development or refinement by offering well-curated datasets that can be used to test and improve the in silico methods. For several decades the main focus of research in the field of natural compounds has been on pharmacodynamics, mostly with in vitro or animal experiments. It is widely known that there are often wide gaps between such studies and human clinical data. This may be, to a good extent, the consequence of ignoring ADME data. The very cheap computational tools and methods allow for not only better insights into the mechanistic aspects of such discrepancies but also better drug development based on natural compounds. The increasingly wider use of generative AI models will expand the design of natural product-inspired derivatives with optimized ADME properties. The mechanistic insights into structure–activity relationships specific to natural compounds provided by certain in silico tools, such as QSAR, pharmacophore models, PBPK models, and even ligand docking and MD and MM simulations, will ultimately intensify the translation of natural compounds’ diversity into life-saving or life-improving medicinal products. One should never forget that such models always have limitations, but with improved experimental validation and better algorithms and computational power, the future is projected to be brighter. For the computational chemist, the drug developer, and the phytochemist, these are exciting times to be alive.

## Figures and Tables

**Table 1:** Natural compounds whose stability/reactivity were evaluated using quantum methods.

Chemical Compound	Stability/Reactivity *	QM Method Used	Reference
Coriandrin	High molecular stability	PM6	[26]
Alternamide A	Highly reactive	PM3	[25]
γ-elemene	Least stable among the six terpene compounds evaluated	MNDO	[27]
Adenine	Moderately reactive	PM6	[26]
Adenosine	Moderately reactive (more reactive than adenine)	PM6	[26]
Coriandrone B	Moderately stable	PM6	[26]
Menthone	Most stable among the six terpene compounds evaluated	MNDO	[27]
Dihydrocoriandrin	Relatively high stability	PM6	[26]
Tryptophan	Relatively high stability	PM6	[26]
β-caryophyllene	Somewhat instable	MNDO	[27]
β-caryophyllene oxide	Somewhat stable	MNDO	[27]
Eucalyptol	Very stable	MNDO	[27]
Pulegone	Very stable	MNDO	[27]

* Theoretical predictions based on QM, not confirmed experimentally.

**Table 2:** QSAR models for the prediction of the oral absorption of pharmaceuticals.

Type of Model	Data Set Size (Training, Test Sets)	Performance (Best Model)	Outcome Variable	Reference
Regression QSAR	86 (67, 9, 10 *)	RMS—9.4% HIA units (training), 19.7% HIA units (CV), 16.0% HIA units (external set)	Human intestinal absorption (%)	[136]
Hologram QSAR, regression	638 (50, 128)	R^2^—0.79, Q^2^—0.63	Human intestinal absorption (%)	[137]
Classification	272 (232, 40)	Accuracy (train set)—71%, accuracy (test set): 60%.	Bioavailability data in healthy human subjects (4 classes of bioavailability: class 1 (<20%), class 2 (20–49%), class 3 (50–79%), class 4 (80–100%).	[138]
Regression and classification models	458	Regression: R^2^—0.60 Classification: CCR—0.88, MCC—0.75 (10-fold cross-validation)	Human intestinal absorption (%). Three ordinal classes of absorption (class 1—>80%, class 2—30–80%, class 3—<30%).	[139]
Regression models based on Abraham descriptors	169 (38 + 131; 31 + 138)	0.85 (train set); 0.78 (cross-validation)	Human intestinal absorption (%)	[140]
Regression and classification	96 (67 + 9 + 12 *)	RMS—6.5 (train set), 27.7 (test set), 22.8 (external prediction set). For classification, sensitivity 100%, specificity 50%.	Human intestinal absorption (%). For classification purposes, a 50% HIA threshold was used to define two classes.	[139,141]
Classification QSAR, using structural descriptors	1262 (899 + 362)	AAE—0.12 (12%); Accuracy: 79–86%	Human intestinal absorption (%, divided in six classes of about 16% per class)	[142]
Regression models using five classes of descriptors	169 (113 + 56)	R^2^—0.86 (training set), 0.73 (test set)	Human intestinal absorption (%)	[143]
Regression model using descriptors computed based on DFT	241 (38 + 203)	RMSE—12.8 (% HIA) (15 on the entire test set)	Human intestinal absorption (%)	[144]
Classification QSAR using a variety of descriptor classes	141 (+ an external data set of 27 compounds)	Accuracy: 88.9% (external data set), 65.71% (10-fold CV)	Human intestinal absorption (%) (5 classes)	[145]
MI-QSAR (QSAR based on “descriptors explicitly derived from simulations of solutes [drugs] interacting with phospholipid membrane models”)	188 (164 + 24)	R^2^ = 0.68 (train set), 0.65 (test set).	Human intestinal absorption (%)	[146]
Regression and classification models (using a variety of descriptor classes)	553 (455 +98)	R^2^—0.76 ** (train set), R^2^—0.79 ** (test set), AME ^a^—7.3% (test set), Accuracy > 96.8%.	Human intestinal absorption (%)	[147]
Multiple regression models using a variety of descriptors	552 (380 + 172)	R^2^—0.64 ** (train set), R2—0.79 ** (test set)	Human intestinal absorption (%)	[148]
Regression models using descriptors computed with two commercial products and predicted pKa	567 (+25 + 22 ***)	R^2^ for log P_eff_ ^b^—0.72–0.84; RMSE—0.35–0.45 log units (equivalent to 2.24–2.82%)	Human intestinal absorption (%)	[149]
Classification QSAR using multiple classification algorithms and 166 descriptors	225 (158 + 67)	Accuracy—94% (training set), 91% (test set) ^c^. κ statistic—0.58	Human intestinal absorption (%). Two classes: high (>30%) and low (<30%).	[150]
Classification QSAR using FP4 and MACCS fingerprints	578 (480 + 98, (+634 ***)	Accuracy—98.5% (training set), 98.8% (test set), 94% (validation set)	Human intestinal absorption (%). Two classes: high (>30%) and low (<30%).	[151]
Regression and classification QSAR using topological descriptors (computed with the CODES program)	367 (202 + 165) ^d^	R^2^ = 0.93 (train set), Q^2^ = 0.92 (LOO cross-validation). Global accuracy: 74%.	Human intestinal absorption (%). Three classes (cut-offs: 30%, 50% and 70%).	[152]
Classification and regression QSAR models build with different descriptors and algorithms	577 (78 + 489)	Accuracy: 99.37%, 99.58% (train set), 95.92%, 94.90% (test set). RMSE—6.39 (train set), 5.71 (test), R^2^—0.972 (train set), 0.953 (test set)	Human intestinal absorption (%). Two classes, using a 30% threshold.	[153]
Regression QSPR models using 2D and 3D descriptors	1272 (1017 + 255)	R^2^ = 0.97, Q^2^ = 0.83, RMSE CV = 0.31 (training test), R^2^ = 0.81, RMSE T = 0.31 (test set)	Caco-2 cell permeability (permeability coefficient of Caco-2 monolayer cell—Papp)	[154]
Classification and regression QSAR/QSPR models	141 (98, +43)	Accuracy: 0.77 (10-fold CV), 0.70 (external data set) R^2^: 0.38 (training set), 0.05 (external data set)	Human intestinal absorption (%). Two classes, using an 85% threshold.	[155]
Regression QSAR using a variety of descriptors computed with the Dragon software	160 (90 + 30 + 40)	R^2^—0.771 (training set), 0.716 (test set). RMSE—0.182 (training set), 0.189 (test set)	Human intestinal absorption (%)—more precisely, log10 (HIA% + 10).	[156]
Regression QSAR using artificial neural networks	86 (67 + 9 + 10)	R^2^—0.802 (test set); RMS—0.59 (train set), RMS—0.42 (test set).	Human intestinal absorption (%).	[157]
Regression QSAR using mainly structural descriptors	467 (417 + 50)	R^2^—0.79 (train set), 0.79 (test set), RMSE—12.3% HIA	Human intestinal absorption (%).	[158]

* Train set, test set, external prediction set. ** The authors have reported the r value (not r^2^). *** External validation data set. ^a^ AME—absolute mean error. ^b^ In vivo human jejunal permeability coefficients. ^c^ The accuracy is relatively high, but the data set was highly imbalanced (balanced accuracy would have been preferable). ^d^ The best performing model selected was trained on only 37 compounds.

**Table 3:** Limitations of computational methods used in assessing PK properties of natural compounds, implications, and potential mitigation approaches.

Method	Key Limitations	Impact on PK Predictions	Potential Mitigation
**QM**	High computational costs; limited to small systems; reliance on approximations; short timescales.	Inaccurate modeling or unreasonably long times for large systems (e.g., membranes) or dynamic processes (e.g., drug transformations).	Advanced computational resources, including GPU acceleration; empirical methods for particular properties; QM/MM were pure QM methods are unrealistic.
**QM/MM**	High computational costs; QM/MM boundary artifacts; poor charge transfer with small QM regions; limited sampling.	Unreasonably long times for large systems, errors in binding energies or reaction kinetics due to error cancelation or inadequate sampling.	Larger QM regions; improved boundary treatments; enhanced sampling methods, including coarse graining.
**Ligand Docking**	Limited protein flexibility, ignorance of indued fit; simplified scoring functions; poor solvent treatment; often poor reporting; the docking score is not sufficient to assert the direction of effect (agonist vs. antagonist).	Errors in binding affinity or pose prediction; misidentification of agonists/antagonists.	Flexible or induced-fit docking, better scoring functions, experimental validation.
**Pharmacophore**	No general scoring method; dependence on a set of pre-generated conformations; static models; dependence on good quality ligand–protein crystal structures; no canonic way of building models.	False positives or missed hits due to oversimplified models or incorrect tautomers.	Improved conformation databases, dynamic modeling.
**QSAR**	Overfitting; limited applicability domain; activity cliffs.	Poor generalizability to new compounds; prediction errors for structurally similar compounds.	High quality, high size training data; use of appropriate techniques to control overfitting; use tools to control for activity cliffs, use local models, use cliff-aware descriptors (e.g., 3D, conformational, quantum).
**MD Simulations**	High computational costs; imperfect force fields; short timescales; not well-suited for systems where quantum effects play a prominent role	Errors in predictions; inadequate sampling of conformational states; poor modeling of large systems.	Polarizable force fields, coarse-grained MD, enhanced sampling, use of QM/MM when quantum effects are important.
**PBPK Models**	Overestimation of CYP3A4 TDI; limited non-CYP enzyme data; software customization needs; limitations in the case of proteins; areas with limited experience; limitations in tissue-specific distribution.	Errors in PK predictions; uncertainties in tissue distribution or special populations.	Imaging-based validation (e.g., PET), customized software for non-CYP enzymes.

**Table 4:** Performance of computational methods in predicting the pharmacokinetic properties of natural compounds compared to experimental data.

Method	Example	Experimental Validation	Performance Summary
**Quantum Mechanics (QM)**	Stereoselectivity of nicotine hydroxylation by CYP2A6 [277]	Yes (retrospective), computed (~97%) vs. wet lab (89–94%)	High agreement
	LogP estimation for BBB permeability (e.g., caffeine) [278]	Yes (clinical data, retrospective)	Good match with known BBB-crossing compounds
	DFT used for C-H bond energy at the main metabolic site (e.g., acetic acid) [279]	Yes (vs. experimentally derived bond energy)	Lower bond dissociation energy at main metabolic site confirmed by experimental data as compared with other C-H bond
	Global reactivity of 4-hydroxyisoleucine [280,281]	Indirect (predicted stability vs. independent plasma stability study)	Supported by external experimental data
**Molecular Docking**	Docking of drugs with CYP2D6 variants [280]	Yes, retrospective correlation (R^2^ = 0.81–0.92)	High agreement
	Flavonoids binding to Pgp [281]	Very weak; correlation r = –0.27 to 0.079	Poor correlation despite otherwise claims
	Lignans and flavonoids binding to Pgp [51]	Partial; 2 of 10 flavonoids experimentally confirmed	Partial success (at least 20%)
	Abietane diterpenes binding to Pgp [282]	Yes, for 2 hemisynthesis compounds	Good performance for two tested compounds
**Pharmacophore Models**	URAT1 inhibitors [105]	Yes, 3 flavonoids of 25 hits were active (relatively low potency)	Modest performance
	CYP2D6 inhibitors [110]	Yes; 42% strong, 33% moderate inhibition	High agreement (75% activity in vitro)
	DDIs via CYP1A2, 2C9, and 3A4 enzymes [283]	Yes (vs in vitro results obtained with fluorescence-based P450 microarrays)	32.1–65.5% depending on model and enzyme
	CYP3A4 inhibitors from *Tripterygium wilfordii* [284]	Ye (vs. in vitro enzyme inhibition assays); 3 of 5 predicted were confirmed	Good agreement
	CYP1A2 inhibitors from herbal compounds [285]	Yes; 7 of 12 compounds active	~58% accuracy for a combined approach (docking + pharmacophore models)
**QSAR Models**	COMFA/COMSIA for natural phenolics [163]	Yes; retrospective (r^2^pred = 0.78, 0.70)	Very good agreement
	Intestinal absorption prediction [286]	Yes; 83% predictions within 2-fold of observed values	Comparable to in vitro method
	Drug absorption in rats [287]	Reliability comparable to the Caco-2 and 2/4/A1 cell lines	Very good agreement
**Molecular Dynamics (MD)**	Withaferin-A and withanone membrane permeability [288]	Yes; imaging based on antibody detection confirmed MD predictions	Excellent agreement
	Curcumin and quercetin binding to CYP3A4 and displacing CDK inhibitors [289]	Yes; docking, MD, and IC50 (in vitro)	Excellent agreement in several validation approaches
**PBPK Models**	Oxymatrine dose prediction [290]	Yes; compared to clinical dose	Predicted dose (367 mg TID) aligned with clinical recommendation
	Prediction of DDIs for hyperforin with sedative-hypnotics in human patients [291]	Yes—model predictions compared with known clinical interactions	Close agreement, all predictions within acceptable margin of error
	PK of hydrastine and berberine [292]	Yes—validated against observed clinical data	Close fit to human PK data
	PK of single dose and multiple dose administration of piperine [293]	Yes—validated against actual clinical data	All error values below the two-fold acceptance criterion

**Table 5:** Synthetic comparison of the four main free ADME web-server applications.

Features	SwissADME	pkCSM	ADMETlab 3.0	admetSAR 3.0
Physicochemical properties	12	7 *	21	10
Medicinal chemistry endpoints	10 **	0	20	4 **
Absorption *** endpoints	3 (C)	3 (N)	9 (2N, 7C)	14 (6N, 6C)
Distribution endpoints	1 (C)	4 (N)	9 (3N, 6C)	11 (1N, 12C)
Metabolism endpoints	5 (C)	7 (C)	14 (C)	15 (C)
Excretion endpoints	0	2 (1N, 1C)	2 (N)	4 (2N, 2C)
PAINS included	Yes	No	Yes	No
Batch evaluation/API support	Multiple smiles allowed	Limit to 100 smiles	Input limited to one smile, but API available	Batch prediction allowed for 1000 molecules.
Interpretation help	++	++	+++	++
Uncertainty estimation	No	No	Yes (prediction probabilities for categorical predictions converted into six symbols)	Yes (prediction probabilities for categorical predictions)
Availability	Free	Free	Free	Free

* Water solubility, although included by pkCSM as an “absorption” endpoint, is in fact a physicochemical property. ** Drug likeness metrics have also been included here. *** Some applications includes Pgp under Absorption, others under Distribution. In this table Pgp endpoints were included under absorption. N—numeric, C—categorical. ++—moderate level. +++—very good level.

## Data Availability

Data are contained within the article.

## Abbreviations

The following abbreviations are used in this manuscript:

ADMET	Absorption, distribution, metabolism, excretion, and toxicity
ADME	Absorption, distribution, metabolism, and excretion
QSAR	Quantitative structure–activity relationship
PBPK	Physiologically based pharmacokinetics
PAINS	Pan-assay interference compounds
cLogP	Calculated partition coefficient
LLE	Lipophilicity ligand efficiency
SFI	Solubility forecast index
PFI	Property forecast index
QM	Quantum mechanics
MNDO	Modified neglect of diatomic overlap
AM1	Austin model 1
PMn	Parametric method n
OMn	Orthogonalization-corrected method n
DFTB	Density-functional tight-binding
HF	Hartree–Fock
SCF	Self-consistent field
MPPT	Møller–Plesset perturbation theory
MP	Møller–Plesset perturbation theory
CI	Configuration interaction theory
CC	Coupled cluster
CASSCF	Complete active space self-consistent field
CASPT2	Complete active space perturbation theory
MCSCF	Multi-configurational self-consistent field
DMRG	Density matrix renormalization group method
DFT	Density functional theory
DFT-D	Dispersion-corrected DFT
GGA	Generalized gradient approximation
MM	Molecular mechanics
OTC	Organic cation transporter
IUPAC	International Union of Pure and Applied Chemistry
SBP	Structure-based pharmacophore
PDB	Protein Data Bank
OATn	Organic anion transporter n
URAT1	Urate transporter 1
CoMFA	Comparative Molecular Field Analysis
HQSAR	Hologram QSAR
PAMPA	Parallel artificial membrane permeation assay
QSPR	Quantitative structure-property relationship
RMS	Root mean square
HIA	Human intestinal absorption
CV	Cross-validation
CCR	Correct classification rate
MCC	Matthews correlation coefficient
AAE	Average Absolute Error
RMSE	Root mean square error
AME	Absolute mean error
BBB	Blood—brain barrier
DMPC	Dimyristoylphosphatidylcholine
EGCG	Epigallocatechin gallate
MD	Molecular dynamics
MM- PBSA	Molecular mechanics Poisson–Boltzmann surface area
MM- GBSA	Molecular mechanics generalized born surface area
CADD	Computer—aided drug design
Kp	Skin permeation coefficient
PK	Pharmacokinetics
Smol	Solvent—accessible molecular surface
SASA	Solvent—accessible molecular surface
Vmol, hfob	Total volume of molecules enclosed by solvent-accessible molecular surface
log Swat	Logarithm of aqueous solubility
QPlogPo/w	Predicted octanol/water partition coefficient
logKhsa	Logarithm of predicted binding constant to human serum albumin
log B/B	Logarithm of predicted blood/brain barrier partition coefficient
BIP caco2	Predicted apparent Caco–2 cell membrane permeability
MDCK	Madin—Darby Canine Kidney
QPMDCK	Apparent MDCK cell permeability
Indcoh	Index of cohesion interaction in solids
Glob	Globularity descriptor
QPpolrz	Predicted polarizability
VDss	Volume of distribution at steady state
HLM	Human liver microsomal stability
RLM	Rat liver microsomal stability
CLp	Plasma clearance
CLr	Renal clearance
MRT	Mean retention time
AUC	Area under the curve
DMPNN	Deep message passing neural networks
nHA	Number of hydrogen acceptors
nHD	Number of hydrogen donors
nRot	Number of rotatable bonds
nRing	Number of rings
MaxRing	Number of atoms in the largest ring
nHet	Number of heteroatoms
fChar	Formal charge
nRig	Number of rigid bonds
FLuc	Firefly luciferase
PPB	Plasma protein binding
